# Association of 24-h Energy Intake Behavior With Depressive Symptoms: Findings From the National Health and Nutrition Examination Survey

**DOI:** 10.1155/da/5544651

**Published:** 2025-04-15

**Authors:** Jiahui Yin, Minqing Gu, Yong Zhou, Yongjun Wang, Min Zhang, Yao Yang, Yiyun Cai, Shen He, Daihui Peng

**Affiliations:** ^1^Department of Chinese Medicine, The University of Hong Kong Shenzhen Hospital, Shenzhen, China; ^2^School of Chinese Medicine, LKS Faculty of Medicine, The University of Hong Kong, Hong Kong, China; ^3^Department of Psychiatry, Kangci Hospital of Jiaxing, Jiaxing, China; ^4^Department of Clinical Nutrition, The First Affiliated Hospital of Shandong First Medical University and Shandong Provincial Qianfoshan Hospital, Jinan, China; ^5^Division of Mood Disorders, Shanghai Mental Health Center, Shanghai Jiao Tong University School of Medicine, Shanghai, China

**Keywords:** breakfast, circadian rhythm, depressive symptoms, energy intake behavior, lunch, National Health and Nutrition Examination Survey

## Abstract

**Objective:** Appetite changes are a significant clinical feature of depression, with circadian rhythms disruption being a crucial mechanism in depression. However, the specific role of eating rhythms in depression remains unclear. This study aimed to assess the relationship between energy intake rhythmicity and depressive symptoms.

**Methods:** A total of 34,974 noninstitutionalized individuals were recruited from the National Health and Nutrition Examination Survey. To investigate the relationship between 24-h energy intake and depressive symptoms, covariate-adjusted sample-weighted regressions were employed. The study analyzed various aspects of energy intake rhythmicity, including the proportion of energy intake from non-meals and meal times, as well as the proportion of energy intake across meals and various time periods (morning, midday, afternoon, evening, night, and overnight).

**Results:** A high proportion of energy intake from non-meals (adjusted odds ratio [OR] = 1.11, 95% confidence interval [CI]: 1.08–1.15) was associated with higher depressive symptoms. The proportion of breakfast energy intake in total daily energy intake was nonlinearly associated with depressive symptoms. In participants with a breakfast energy intake below 20%, the prevalence of depressive symptoms decreased by 15% (adjusted OR = 0.85, 95% CI: 0.75–0.96) per each 10% increment in the proportion of breakfast energy intake. A U-shaped relationship was identified between the timing of the day's last energy intake and depressive symptoms, with the lowest prevalence occurring at 7:48 PM (before 7:48 PM: adjusted [OR = 0.96, 95% CI: 0.94–0.98]; after 7:48 PM: adjusted [OR = 1.11, 95% CI: 1.03–1.20]).

**Conclusions:** Among adults in the United States, the proportion of energy consumed from non-meals was associated with higher depressive symptoms. Adequate energy intake at breakfast and moderate end-times of energy intake were linked to reduced depressive symptoms. These results may contribute to designing of future studies on dietary rhythm interventions for managing depression.

## 1. Introduction

Circadian disruptions contribute to various psychiatric and neurodegenerative diseases [1]. Moreover, circadian disruption has also been widely observed in patients with major depressive disorder (MDD) [2]. The disruption of biological rhythms can also contribute to the onset and recurrence of MDD [3]. Eating rhythmicity is a significant component of the circadian timing system. Current research on the rhythm of depression focuses mainly on sleep rhythmicity, with insufficient attention being paid to eating rhythmicity.

Change in appetite is a defining feature of patients with MDD. The reward system is believed to play a role in appetite changes in patients with depression [4]. Patients with MDD and an abnormal appetite exhibit marked immunometabolic dysregulation [5]. The study of eating patterns has translational significance in the prevention and treatment of depression.

Current research has highlighted the positive effects of nutrients on depression [6], including omega-3 fatty acids [7], vitamin D [8], and dietary fiber [9]. Nutrients can influence depression through mechanisms including inflammation, oxidative stress, the hypothalamic–pituitary–adrenal axis, tryptophan–kynurenine metabolism, neurogenesis, brain-derived neurotrophic factor, mitochondrial dysfunction, gut microbiota, obesity, and epigenetics [10]. Eating rhythmicity can exert hormonal, neurobiological, and microbiome effects [11, 12]. Moreover, modifying eating rhythmicity incurs lower economic costs than changing the diet content. Studies have discovered that controlling eating rhythmicity can lower fasting glucose levels, reduce insulin resistance [13], promote weight loss [14], improve cardiometabolic health [15] and enhance mood [16]. However, the effect of eating rhythmicity on depression remains unclear.

Dashtiet et al. identified a causal link between skipping breakfast and increased depressive symptoms using Mendelian randomization [17]. One study demonstrated that eating at night could increase depression among shift workers. Qian et al. [18] reported that simulated nighttime work combined with both daytime and nighttime eating led to elevated depression-like mood levels. Some studies have also identified that healthy eating habits (e.g., eating lunch and dinner) [19, 20] are associated with a reduced prevalence of depression. However, these studies mainly focused on lifestyle habits and did not systematically consider eating rhythmicity. Therefore, the relationship between all-day eating rhythmicity and depression warrants further investigation.

To examine the relationship between 24-h energy intake rhythmicity and depressive symptoms in adults, we compared the 24-h energy intake of participants with and without depressive symptoms using a nationally representative sample from the National Health and Nutrition Examination Surveys (NHANES). We analyzed the relationship between different 24-h energy intake patterns and depressive symptoms.

## 2. Materials and Methods

### 2.1. Participants

A cross-sectional analysis of the NHANES data from 2005 to 2018 was conducted. The National Center for Health Statistics (NCHS) conducts a nationally representative NHANES survey that uses multistage, stratified probability cluster sampling to evaluate the nutritional status and general health of the US noninstitutionalized population. The NHANES study protocol was approved by the NCHS Research Ethics Review Board, and the participants provided signed informed consent at the time of recruitment. This study was conducted in accordance with the Strengthening the Reporting of Observational Studies in Epidemiology Reporting Guidelines.

The inclusion criteria were applied to participants based on the following eligibility requirements: (1) age of 18 years or older and (2) provision of at least one 24-h dietary recall during either an in-person session or follow-up phone interview. The exclusion criteria were as follows: (1) incomplete Patient Health Questionnaire-9 (PHQ-9) and (2) missing energy intake data from both 24-h dietary recalls.

### 2.2. Measures

#### 2.2.1. Energy Consumption Assessment

Dietary information was collected through two 24-h dietary recalls: the first in person, and the second via phone wasconducted 3–10 days later. The NHANES mobile examination center's private room served as the venue for in-person interviews. Dietary recall data were collected using the Automated Multiple-Pass Method (AMPM), developed by the US Department of Agriculture (USDA) [21]. The AMPM is a fully computerized recall method designed to provide an efficient and accurate approach for large-scale national surveys. The AMPM employs a structured five-step interview process summarized as follows:

1. Quick list: Participants list all foods and beverages consumed from midnight to midnight on the previous day.
2. Forgotten foods: Participants are prompted about commonly forgotten foods or beverages to ensure completeness.
3. Time and occasion: The times of consumption and the occasion (e.g., breakfast and lunch) are recorded for each item.
4. Detailed cycle: Detailed descriptions, portion sizes, and additions for each food item are collected. Interviewers also probe for any food or beverage consumed between meals.
5. Final probe: Participants are asked if they remember any additional foods or beverages not previously mentioned.

The AMPM includes a comprehensive compilation of standardized food-specific questions, with potential responses routed based on prior answers. Additionally, the AMPM is updated annually to reflect changes in the food supply and meet the needs of the research community. The AMPM has been validated in multiple studies and proven effective for accurately collecting dietary intake data in adults [22–24]. During the study, participants were provided with a set of measuring tools in the dietary interview room, including a ruler, different mugs, glasses, drink boxes, bowls, bottles, measuring cups and spoons, household spoons, bean bags, thick sticks, and circles to assist when reporting food quantities. Once the in-person interview concluded, the participants were provided with a ruler, spoons, measuring cups, and a food model booklet containing two-dimensional illustrations of the available measuring guides. These items were utilized to record the amount of food consumed during telephone interviews. In addition, the dietary assessment in this study was designed and implemented by professional teams to ensure high reliability. The dietary interview component, referred to as What We Eat in America (WWEIA), is a collaborative initiative between the USDA and the US Department of Health and Human Services (DHHS). Within this collaboration, the NCHS at DHHS oversees survey design and data collection, meanwhile, the Food Surveys Research Group at the USDA manages dietary data collection methodology, database maintenance, and data review and processing.

Participants reported the exact time, including both hours and minutes of their meals, during a 24-h dietary recall. Based on these data, our analysis results were precise to the minute. Regarding the synchrony between eating and nutrient intake, NHANES provides nutrient data directly linked to reported eating times based on the Food and Nutrient Database for Dietary Studies (FNDDS) from the USDA. This comprehensive nutrient database processes dietary intake information to generate detailed nutrient data for each time point, which was then used to analyze the synchrony between eating times and nutrient intake. Based on the data gathered, we calculated the total energy intake over a 24-h period as well as the energy intake at various intervals throughout the day.

#### 2.2.2. Depressive Symptoms

In this study, the nine-item PHQ-9 was employed to assess depressive symptoms. The PHQ-9 is widely recognized as an accurate and reliable tool for depression screening. The total score ranges from 0 to 27, with high values reflecting severe depression. Analyses used a PHQ-9 cutoff score of 10 or higher to identify clinically significant depressive symptoms, yielding 85% sensitivity and 89% specificity for MDD when compared with clinical interview criteria [25].

#### 2.2.3. Covariates

We selected covariates based on the existing literature that may be related to energy intake rhythmicity and the theoretical frameworks, ensuring they were not on the causal pathway connecting the two [20, 26–30]. Age, race, ethnicity, sex, occupational status, family income, marital status, educational level, physical activity, drinking and smoking behaviors, the Healthy Eating Index (HEI), duration of sleep, body mass index (BMI), comorbid conditions, dietary recall day of the week, and diurnal total energy intake were among the covariates considered.

Furthermore, we evaluated race and ethnicity using self-reported data. The participants were divided into the following five racial and ethnic groups: Mexican American, non-Hispanic Black, non-Hispanic White, other Hispanic, and other (including multiracial). We classified family income into three categories based on the family poverty income ratio, as employed by US government agencies to provide NHANES food and health data: low income (≤1.3), medium income (>1.3–3.5), and high income (>3.5) [31]. Three categories of educational attainment were established as follows: less than high school, completed high school, and beyond high school. Physical activity was evaluated based on the participants' reported levels of vigorous (e.g., high-intensity workouts, basketball, and running) and moderate activities (e.g., brisk walking, swimming, or cycling at a normal pace). Smoking status was categorized into three groups: never smoked (less than 100 cigarettes), former smoker (smoked >100 cigarettes but not currently), and current smoker (smoked >100 cigarettes and currently smoking daily or occasionally). Drinking status was classified into four categories: never (less than 12 drinks in a lifetime), former (>12 drinks in a single year but none in the previous year, or >12 drinks in a lifetime), light/moderate drinker (less than one drink per day on average for women or less than two drinks per day on average for men), and heavy drinker (>1 drink per day on average for women or >2 drinks per day on average for men). Participants were classified as having comorbid conditions if they reported at least one of the following: diabetes, renal failure, stroke, kidney stones, heart failure, liver disease, rheumatoid arthritis, or cancer. The HEI is a tool used to assess the alignment of an individual's diet with established dietary guidelines, offering a single score that reflects the overall dietary quality [32]. The total nutrient intake over the 24 h preceding the in-person interview was used to compute the HEI. The average of two 24-h recalls served as the basis for total energy intake. If only one 24-h recall was performed, the total energy consumption for that day represented the total energy intake. The detailed definitions of the covariates are provided in Table S1.

### 2.3. Process of Extracting Data

We initiated the search by accessing the NHANES database for 24-h dietary recall interviews and PHQ-9 questionnaire data, available from the 2005 to 2018 surveys, using the official NHANES search portal [33]. Subsequently, covariate information for participants from the same survey years was identified, and the corresponding download links were located on the NHANES website. The relevant data files were then downloaded locally and systematically merged using the unique identification numbers assigned to each participant by the NHANES, ensuring accurate alignment across datasets. The merged dataset was systematically organized and cleaned to ensure accuracy and readiness for analysis. This process included removing inconsistencies, standardizing variables, and ensuring data completeness. All steps in data extraction, cleaning, and analysis strictly adhered to the NHANES protocols [34], ensuring the validity and reliability of our methodology. This rigorous approach allowed us to create a comprehensive and high-quality dataset.

### 2.4. Statistical Analysis

To provide nationally representative estimates, every analysis employed the NHANES complex sample design. For continuous variables, participant characteristics were expressed as means with 95% confidence intervals (CIs), while categorical variables were expressed as percentage frequencies with 95% CIs. The *χ*^2^ test was used to compare categorical data, whereas *t*-tests were utilized to analyze continuous data. As the missing values were all categorical variables, we created dummy variables to account for the missing covariates (missing categories were assigned a separate class).

Logistic regression models were used to compute odds ratios (ORs) and 95% CIs to evaluate the relationship between eating behaviors and depressive symptoms. No adjustments were made to the crude model. Moreover, age, race, ethnicity, sex, occupational status, family income, marital status, educational level, physical activity, drinking and smoking behaviors, BMI, duration of sleep, comorbid conditions, HEI, dietary recall day of the week, and diurnal total energy intake were all considered when creating the adjusted model.

To investigate the nonlinear relationship between continuous variables and depressive symptoms, we employed a restricted cubic spline (RCS) approach with five knots at the 5^th^, 28^th^, 50^th^, 73^rd^, and 95^th^ percentiles using the inflection point as the reference. A recursive approach was utilized to identify the inflection point of the exposure, which included selecting the inflection point along the predefined interval and the one that generated the maximum likelihood model. Based on the RCS identification of the inflection points, segmented ORs and 95% CIs were computed for each side.

Statistical analyses were conducted using R version 4.2.0 (https://cran.r-project.org/). In every analysis, a double-tailed *p* − value of less than 0.05 was deemed statistically significant.

## 3. Results

### 3.1. Characteristics of the Participants

This study included 34,974 participants (Figure S1). Table S2 lists the characteristics of excluded and included participants. Approximately, 3025 individuals (8.01%; 95% CI, 7.41%–8.61%) were identified as having depressive symptoms based on their PHQ-9 score (Table 1). The univariate analysis results are displayed in Table S3, whereas the missing variables are listed in Table S4.

**Figure 1:**
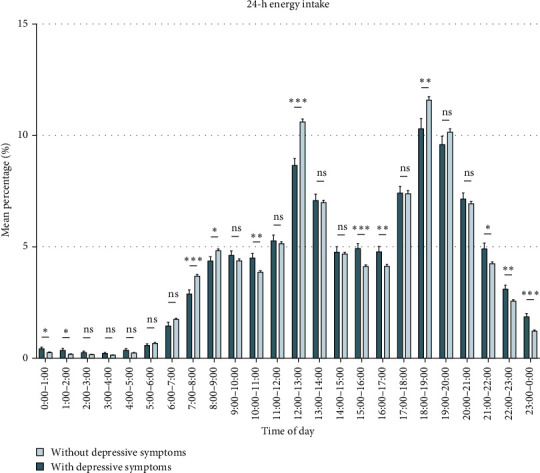
Mean percentage of 24-h energy intake by hour among participants with or without depressive symptoms. Error bars represent the standard error of the mean. Time was divided into hourly intervals, spanning from the beginning of the hour to the end of the hour. For example, 0:00–1:00 indicates the period from 0:00 to just before 1:00. *⁣*^*∗*^*p* < 0.05, *⁣*^*∗∗*^*p* < 0.01, *⁣*^*∗∗∗*^*p* < 0.001.

Figure 1 (Table S5) compares the energy intake of participants with and without depressive symptoms. We discovered that during non-meal periods and at night (21:00–0:00, 0:00–1:00, 10:00–11:00, 15:00–17:00), participants with depressive symptoms consumed more energy than those without depressive symptoms. During 7:00–9:00, 12:00–13:00, and 18:00–19:00, participants without depressive symptoms consumed more energy than those with depressive symptoms. Based on the differences in 24-h energy intake between participants with and without depressive symptoms and previous literature (Table S5), we summarized the rhythmic characteristics of energy intake that may be associated with depressive symptoms (Table S6) and validated them in subsequent analyses.

The distribution of the variables of interest (occupational status, income level, BMI, physical activity, and energy intake level) across different energy intake behaviors were compared. We discovered that participants who began eating at or after 9:00 AM were less physically active, more likely to be in a “Looking for work” or “Not working” status, had low economic status, a high proportion with BMI ≥30.0 or BMI <18.5 kg/m^2^, and tended to have low total energy intake. Among the participants whose eating ended at or after 8:00 PM, a high proportion had a BMI <18.5 kg/m^2^ and they tended to have a high total energy intake. Participants with high non-meal energy intake were more likely to have a poor economic status and a high total energy intake. The detailed analysis results are presented in Tables S7–S12.

### 3.2. Association between 24-Hour Energy Intake Behaviors and Depressive Symptoms

#### 3.2.1. Overall Characteristics

**Figure 2:**
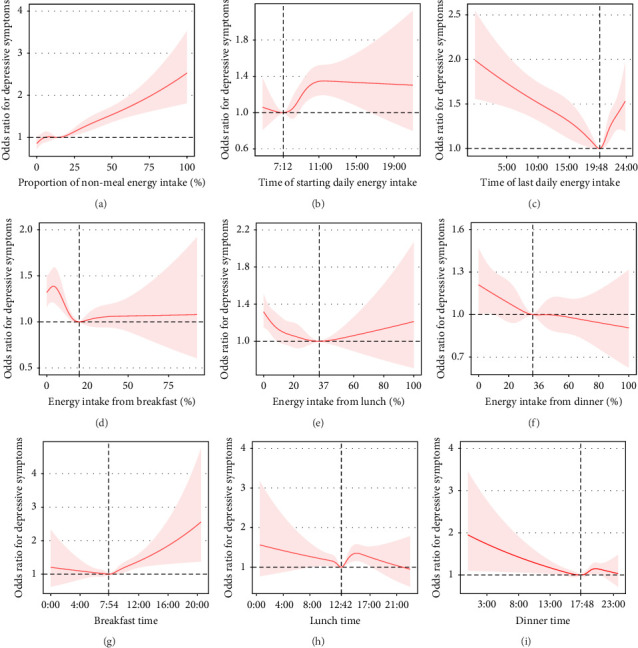
Restricted cubic spline of the relationship between 24-h energy intake behaviors and despressive symptoms. Odds ratios (solid lines) and 95% CIs (shaded areas) were adjusted for age, sex, race and ethnicity, education level, marital status, family income, body mass index, smoking status, drinking status, physical activity, comorbid condition, sleep duration, total energy intakes, healthy eating index, first dietary recall day of the week, and second dietary recall day of the week. Vertical dotted lines indicate the cut point. (A) The proportion of nonmeal energy intake was positively linearly associated with depressive symptoms. (B) The inflection point for the time of starting daily energy intake is at 7:12 AM. (C) The inflection point for the time of the last daily energy intake is at 19:48 (7:48 PM). (D) The inflection point for energy intake from breakfast is at 20%. (E) The inflection point for energy intake from lunch is at 37%. (F) The inflection point for energy intake from dinner. (G) The inflection point for breakfast time is at 7:54 AM. (H) The inflection point for lunchtime is at 12:42 PM. (I) The inflection point for dinner time is at 17:48 (5:48 PM).

1. The proportion of daily energy intake from non-meals was associated with depressive symptoms (adjusted OR = 1.11, 95% CI: 1.08–1.15) (Table 2, Figure 2A).
2. The time of starting daily energy intake demonstrated a nonlinear association with depressive symptoms (P for nonlinearity = 0.053). For participants who started eating after 7:12 AM, the prevalence of depressive symptoms increased by 5% (adjusted OR = 1.05, 95% CI: 1.02–1.09) with each 1-h delay in start time (Figure 2B).
3. The time of last daily energy intake was nonlinearly associated with depressive symptoms. The prevalence of depressive symptoms decreased by 4% (adjusted OR = 0.96, 95% CI: 0.94–0.98) per each 1-h increment in the time of last daily energy intake in participants with a time of last daily energy intake before 7:48 PM. For participants with a time of last daily energy intake after 7:48 PM, the prevalence of depressive symptoms increased by 11% (adjusted OR = 1.11, 95% CI: 1.03–1.20) per each 1-hour delay in the time of last daily energy intake (Figure 2C).
4. Based on the analysis of energy intake periods, we identified a saturation point (23%) of energy intake proportion from 5:00–9:00 AM in relation to the depressive symptoms (below 23%, adjusted OR = 0.84, 95% CI: 0.75–0.94; above 23%, adjusted OR = 1.17, 95% CI: 0.98–1.41). Energy intake between 11:00 PM–5:00 AM was positively associated with depressive symptoms (adjusted OR = 1.17, 95% CI: 1.10–1.24). However, energy intake from 12:00 AM–1:00 PM was negatively associated with depression (adjusted OR = 0.85, 95% CI: 0.75–0.96) (Table S13).
5. We also analyzed the relationships among other circadian rhythms, energy intake behaviors, and depressive symptoms. We noted that participants with more than 1 h of recreational physical activity per week had a lower prevalence of depressive symptoms than those with activity less than 1 h per week. We also discovered that the occupational status of “Looking for work” or “Not working” was associated with depressive symptoms. Additionally, participants with zero hours of work per week had a higher prevalence of depressive symptoms than those with more than zero hours of work per week. We found that participants who started energy intake at or after 9:00 AM, those who had breakfast at or after 9:00 AM, and participants whose lunch energy intake accounted for 17.1% or less of their total daily energy intake were more likely to be in a “Looking for work” or “Not working” status. Additionally, these participants were likely to report zero working hours per week. Furthermore, we demonstrated that participants who started energy intake at or after 9 : 00 AM and those who had breakfast at or after 9:00 AM had short durations of recreational physical activity.
6. In addition, we employed the HEI to assess the nutritional components and dietary quality. High HEI scores were associated with a low prevalence of depressive symptoms. Participants who had breakfast at or after 9:00 AM or who started energy intake at or after 9:00 AM had a low HEI. Participants whose breakfast energy intake accounted for more than 13.3% of their total daily energy intake had elevated HEI.

#### 3.2.2. Breakfast

1. The proportion of breakfast energy intake in total daily energy intake was nonlinearly associated with depressive symptoms. In participants with a breakfast energy intake below 20%, the prevalence of depressive symptoms decreased by 15% (adjusted OR = 0.85, 95% CI: 0.75–0.96) per each 10% increment in the proportion of breakfast energy intake (Table 3, Figure 2D).
2. Breakfast time was approximately nonlinearly associated with depressive symptoms (before 7:54 AM, adjusted OR = 0.97, 95% CI: 0.85–1.10; after 7:54 AM: adjusted OR = 1.09, 95% CI: 1.03–1.15, P for nonlinearity = 0.077) (Table 3, Figure 2G).

#### 3.2.3. Lunch

1. The proportion of lunch energy intake in total daily energy intake was approximately nonlinearly associated with depressive symptoms (*P* for nonlinearity = 0.06). In participants with a lunch energy intake below 37%, the prevalence of depressive symptoms decreased by 9% (adjusted OR = 0.91, 95% CI: 0.86–0.95) per each 10% increment in the proportion of lunch energy intake (Table 3, Figure 2E).
2. Lunchtime was nonlinearly associated with depressive symptoms (P for nonlinearity = 0.002). With the extension of lunchtime before 12:42 PM, the prevalence of depressive symptoms exhibited a decreasing trend (adjusted OR = 0.94, 95% CI: 0.88–1.01); with the extension of lunchtime after 0:42 PM, the prevalence of depressive symptoms had an increasing trend (adjusted OR = 1.04, 95% CI: 0.99–1.10) (Table 3, Figure 2H).

#### 3.2.4. Dinner

1. Participants who reported eating dinner at both dietary recalls had a lower prevalence of depressive symptoms than those who reported skipping dinner at both recalls. However, this association was not significant after adjustment for all covariates (unadjusted OR: 0.63, 95% CI: 0.46–0.87, *p*=0.005; adjusted OR: 0.98, 95% CI:0.67–1.42) (Table S14). After adjusting for all covariates, the associations between the proportion of energy intake at dinner, the proportion of evening (6–8 PM) energy intake, the proportion of night (8–11 PM) energy intake, and dinnertime with depressive symptoms were not statistically significant (Table 3, Figure 2F).
2. The proportion of nighttime (11 PM to 5 AM) energy intake to the total daily energy intake was positively associated with depressive symptoms. After adjusting for all covariates, participants who reported nighttime energy intake had a 42% increased prevalence of depressive symptoms compared with those who did not consume energy at night (unadjusted OR: 1.65, 95% CI: 1.45–1.86, *p*  < 0.001; adjusted OR = 1.42, 95% CI: 1.23–1.63, *p*  < 0.001) (Table 3, Figure 2I).

## 4. Discussion

This nationally representative cross-sectional study identified correlations between different energy intake patterns and depressive symptoms in US adults. We discovered that the proportion of energy consumed from non-meals was associated with high depressive symptoms, while adequate energy intake at breakfast and a moderate end time of eating were associated with few depressive symptoms. To the best of our knowledge, this is the first study to systematically demonstrate the associations between 24-h energy intake behaviors and depressive symptoms.

Our study replicated and broadened previous findings in multiple ways. First, we replicated prior evidence confirming that regular breakfast [17] and lunch [19, 20] were associated with few depressive symptoms, and the proportions of non-meal energy intake and nighttime energy intake were associated with high depressive symptoms [35, 36]. We extended the existing knowledge by demonstrating that adequate energy intake at breakfast and lunch (breakfast: >20% of 24-h intake, lunch: >37% of 24-h intake), along with moderate start (before 7:12 AM), and end energy intake time (7:48 PM) were associated with few depressive symptoms. In their scaled analysis of Australian adult dietary intake data, Wilson et al. [27] discovered that individuals following a late pattern (skipping or delaying breakfast and consuming more in the evening) had an increased prevalence of mood disorders, whereas those adhering to a traditional pattern (main meals at breakfast, lunch, and dinner) had a decreased prevalence of first-episode mood disorders. We were interested in the kind of energy intake rhythmicity associated with depressive symptoms; therefore, we directly analyzed the relationship between various 24-h energy intake behaviors (timing of meals, proportion of energy intake at meals, etc.) and depressive symptoms.

We also explored the mechanisms underlying the association between energy intake rhythm and depressive symptoms. Specifically, we aimed to explore the influence of other rhythms, including work hours and the duration of recreational physical activity, on the relationship between energy intake rhythms and depressive symptoms. We noted that participants who had breakfast at or after 9:00 AM, started energy intake at or after 9:00 AM, or whose lunch energy intake constituted 17.1% or less of their total daily energy intake were more likely to be unemployed and experience depressive symptoms. Unemployed participants tended to exhibit depressive symptoms and display abnormal energy intake rhythms. Additionally, we observed that participants who started energy intake at or after 9:00 AM and those who had breakfast at or after 9:00 AM had short durations of recreational physical activity. Participants who engaged in recreational physical activity tend to start energy intake earlier in the day and are less likely to experience depressive symptoms. Additionally, we investigated the role of dietary composition in this relationship. The analysis revealed that participants who had breakfast at or after 9:00 AM or who started energy intake at or after 9:00 AM had low dietary quality, as measured by the HEI, and were likely to exhibit depressive symptoms. Conversely, the participants whose breakfast energy intake exceeded 13.3% of their total daily calories had elevated HEI and were less prone to depressive symptoms. A previous study also revealed that early time-restricted eating can alter dietary quality [37]. Energy intake rhythms may influence depressive symptoms by affecting dietary quality. However, this study demonstrated an independent association between energy intake rhythms and depressive symptoms rather than a spurious correlation caused by confounding factors, including work, physical activity, BMI, and marital status, after adjusting for these confounders.

Bidirectional relationships between energy intake rhythmicity and depression are plausible. An altered appetite is a defining feature of depression. Depression is genetically correlated with circadian rhythms [38, 39], and patients with depression have alterations in brain areas, such as the striatum [40], which may lead to altered energy intake rhythmicity. Energy intake rhythmicity may play a role in promoting or preventing depression onset. Interventions targeting eating rhythms have been demonstrated to attenuate the negative consequences of sleep disruption in rodents [41, 42], enhance neuronal function and autophagy in drosophila [43], reduce levels of inflammation [44, 45] and oxidative stress [14, 46, 47], increase the abundance of gut flora [48, 49], and promote weight loss [50] in humans. Eating rhythms may impact depressive symptoms through these pathways.

Some studies have also identified that short-term dietary rhythmicity interventions (time-restricted eating) can improve participants' moods [16, 18], while long-term studies targeting dietary rhythmicity remain inadequate. The presentation of energy intake rhythmicity identified in this study can be targeted in future experimental trials to determine whether modifying energy intake rhythmicity influences depression.

### 4.1. Strengths and Limitations

This study has several advantages. First, we utilized data obtained from the NHANES Survey conducted by the Centers for Disease Control and Prevention, which examined the nutritional status and general health of a non-institutionalized US population. The FNDDS of the United States Department of Agriculture served as the foundation for detailed energy intake calculations made by the NHANES, which utilized 24-h meal recalls to gather information on US adults' dietary consumption. Consequently, the study's data enabled a precise assessment of the participants' energy consumption throughout 24 h. Second, as the NHANES employs a stratified, multistage probability cluster sampling approach, our results can be generalized to a large population. The participants included in this study reflect roughly 17.6 million individuals with depressive symptoms and 201.8 million without such symptoms across the United States. Third, our study is the first to compare differences in energy intake rhythmicity between patients with and without depressive symptoms and to systematically assess the relationship between energy intake rhythmicity and depressive symptoms.

The current study has several limitations. First, the cross-sectional nature of the study prevented us from determining the temporal link between energy consumption patterns and depression. Second, individuals with depressive symptoms who were not clinically diagnosed with MDD were also included in the research sample. Further large-scale studies including individuals with MDD are required to extrapolate our findings to this patient population [28]. Third, we employed two nonconsecutive 24-h dietary reviews to assess the participants' energy intake patterns. This assessment method may not fully reflect long-term energy intake patterns, potentially leading to an underestimation of the true relationship between energy intake patterns and depressive symptoms. Finally, unadjusted confounders may exist in the analysis, such as loneliness or social life, which were not evaluated in the NHANES dataset. These factors are linked to depressive symptoms and can potentially influence food intake [51, 52]. Future studies should include these variables to better understand their roles in the association between energy intake patterns and depression.

## 5. Conclusions

The findings of this cross-sectional study indicate that among the US adult population, the proportion of energy consumed from non-meals is associated with increased depressive symptoms, and adequate energy intake at breakfast and a moderate end-time of energy intake are associated with reduced depressive symptoms. The findings of this study may guide the planning of future dietary rhythm intervention studies on depression.

## Figures and Tables

**Table 1:** Characteristics of participants in the NHANES 2005–2018 cycles (*n* = 34,974).

Characteristic^a^	Participants		
	With depressive symtoms	Without depressive symtoms	*p*-value
	(*n* = 3025)	(*n* = 31,949)	
Age			<0.001
18 to <40 years	1046 (37.37) (34.89–39.86)	12,156 (38.75) (37.43–40.07)	—
40 to <60 years	1137 (42.68) (40.25–45.11)	9575 (35.39) (34.51–36.27)	—
≧60 years	842 (19.95) (18.00–21.89)	10,218 (25.86) (24.75–26.97)	—
Sex			<0.001
Female	1918 (62.70) (60.05–65.34)	15,847 (50.27) (49.63–50.91)	—
Male	1107 (37.30) (34.66–39.95)	16,102 (49.73) (49.09–50.37)	—
Race and ethnicity			<0.001
Non-Hispanic White	1268 (62.73) (58.82–66.64)	13,578 (67.76) (65.26–70.27)	—
Non-Hispanic Black	673 (13.68) (11.67–15.69)	6970 (11.07) (9.70–12.43)	—
Mexican American	387 (7.81) (6.07–9.55)	2896 (5.11) (4.37–5.85)	—
Other Hispanic	477 (8.69) (6.83–10.54)	5180 (8.68) (7.36–10.01)	—
Other race/multiple races	220 (7.09) (5.62–8.56)	3325 (7.37) (6.61–8.14)	—
Educational level			<0.001
Less than high school	1076 (24.90) (22.57–27.23)	7534 (14.91) (13.87–15.95)	—
Completed high school	757 (28.24) (25.83–30.66)	7644 (23.82) (22.79–24.86)	—
Beyond high school	1190 (46.86) (43.91–49.80)	16,750 (61.27) (59.58–62.95)	—
Occupational status			<0.001
Working at a job or business	1003 (38.21) (35.52–40.89)	17772 (61.00) (59.92–62.08)	—
With a job or business but not at work	42 (1.16) (0.75–1.56)	687 (2.61) (2.29–2.92)	—
Looking for work	142 (4.41) (3.45–5.36)	1156 (3.09) (2.81–3.37)	—
Not working	1836 (56.23) (53.56–58.89)	12317 (33.30) (32.20–34.40)	—
Family income^b^			<0.001
Low income	1,453 (42.73) (39.56–45.90)	8,831 (20.42) (19.18–21.66)	—
Medium income	930 (35.89) (33.07–38.72)	11,163 (35.07) (33.75–36.38)	—
High income	379 (21.38) (18.61–24.14)	9342 (44.52) (42.63–46.40)	—
Marital status			<0.001
Married/living with partner	1596 (53.14) (50.43–55.86)	12,073 (36.44) (35.16–37.73)	—
Never married/widowed/divorced/separated	1313 (46.86) (44.14–49.57)	18,415 (63.56) (62.27–64.84)	—
Alcohol status			<0.001
Never drinking	377 (9.57) (8.21–10.92)	4,522 (11.42) (10.49–12.35)	—
Former drinker	608 (18.81) (16.63–20.99)	4,692 (12.66) (11.93–13.39)	—
Current light/moderate drinker	1124 (44.93) (41.97–47.88)	14,850 (54.19) (52.76–55.62)	—
Current heavier drinker	730 (26.70) (24.32–29.08)	6027 (21.73) (20.78–22.69)	—
Smoking status			<0.001
Never smoking	1214 (38.90) (35.91–41.88)	17,511 (56.54) (55.41–57.66)	—
Former smoker	642 (20.97) (18.67–23.28)	7451 (24.90) (23.98–25.81)	—
Current smoker	1083 (40.13) (37.15–43.11)	5760 (18.56) (17.78–19.35)	—
Physical activity			<0.001
Inactive	1905 (65.76) (62.69–68.83)	13,663 (43.21) (41.51–44.92)	—
Moderate	519 (22.22) (19.42–25.02)	7129 (28.31) (27.25–29.37)	—
Vigorous	121 (4.17) (2.96–5.37)	2329 (8.79) (8.21–9.38)	—
Both moderate and vigorous	4603 (16.21) (14.92–17.49)	192 (7.85) (6.07–9.63)	—
BMI			<0.001
<18.5 kg/m^2^	66 (2.10) (1.39–2.80)	537 (1.60) (1.39–1.80)	—
18.5 to <25.0 kg (m)^2^	702 (24.88) (22.35–27.42)	8997 (29.22) (28.16–30.27)	—
25.0 to <30.0 kg (m)^2^	775 (25.87) (23.56–28.17)	10,512 (33.14) (32.20–34.07)	—
≥30.0 kg (m)^2^	1441 (47.15) (44.19–50.11)	11,617 (36.05) (34.92–37.18)	—
Sleep duration			<0.001
<6 hr	1682 (55.50) (52.90–58.10)	23,479 (75.60) (74.75–76.44)	—
6 to 8 hr	830 (26.31) (23.75–28.88)	3858 (9.91) (9.38–10.43)	—
>8 hr	490 (18.18) (15.62–20.74)	4543 (14.50) (13.77–15.23)	—
Comorbid condition			<0.001
No	1175 (41.37) (39.12–43.63)	18,448 (59.76) (58.61–60.92)	—
Yes	1840 (58.63) (56.37–60.88)	13,365 (40.24) (39.08–41.39)	—
Total energy intakes			<0.001
Quartile 1 (<1458 kcal)	982 (32.66) (30.03–35.30)	7762 (23.81) (22.78–24.84)	—
Quartile 2 (1458–1916.5 kcal)	805 (26.82) (24.34–29.31)	7937 (24.51) (23.66–25.35)	—
Quartile 3 (1917–2491 kcal)	716 (23.68) (21.74–25.62)	8029 (24.92) (24.16–25.68)	—
Quartile 4 (>2491 kcal)	522 (16.84) (14.82–18.86)	8221 (26.76) (25.52–28.01)	—
HEI			<0.001
Quartile 1 (<40.396)	936 (29.94) (27.72–32.16)	7808 (21.42) (20.68–22.16]	—
Quartile 2 (40.396–49.668)	726 (24.07) (22.05–26.09)	8019 (25.25) (24.54–25.95	—
Quartile 3 (49.669–59.543)	703 (23.58) (21.54–25.62)	8045 (26.31) (25.58–27.04)	—
Quartile 4 (>59.543)	660 (22.41) (20.58–24.25)	8077 (27.02) (26.29–27.76)	—
First dietary recall day of the week			0.02
Weekday	1922 (74.12) (72.09–76.15)	19,558 (71.44) (70.70–72.18)	—
Weekend	1103 (25.88) (23.85–27.91)	12,391 (28.56) (27.82–29.30)	—
Second dietary recall day of the week			0.49
Weekday	2131 (74.63) (72.40–76.86)	22,444 (73.79) (72.64–74.93)	—
Weekend	540 (25.37) (23.14–27.60)	5837 (26.21) (25.07–27.36)	—
PHQ-9 score, mean (95% CI)^c^	14.04 (13.87, 14.20)	2.13 (2.09, 2.17)	<0.001

Abbreviations: BMI, body mass index; HEI, Healthy Eating Index; NHANES, National Health and Nutrition Examination Survey; SE, standard error.

^a^Data are presented as unweighted number (weighted percentage) [95% CI] unless otherwise indicated.

^b^Categorized into the following 3 levels based on the family poverty income ratio: low income (1.3 to 3.5), and high income (>3.5).

^c^PHQ-9 scores range from 0 to 27, with a score greater than 9 used to indicate depressive symtoms in this study.

**Table 2:** Association between 24-h energy intake behaviors and depressive symptoms.

Exposure^a^	Model^b^	Cut points^c^	No.	OR (95% CI)	*p*-value	*p* for nonlinearity
Time of starting daily energy intake	Crude model	Before 7:06 AM	7617	0.94 (0.77, 1.15)	0.533	**<0.001**
		After 7:06 AM	27,357	**1.13 (1.10, 1.16)**	**<0.001**	
	Adjusted model	Before 7:12 AM	7860	1.00 (0.81, 1.23)	0.978	0.053
		After 7:12 AM	27,114	**1.05 (1.02, 1.09)**	**0.005**	

Time of last daily energy intake	Crude model	Before 7:54 PM	16,107	**0.95 (0.94, 0.96)**	**<0.001**	**<0.001**
		After 7:54 PM	18,867	**1.14 (1.06, 1.23)**	**<0.001**	
	Adjusted model	Before 7:48 PM	15,998	**0.96 (0.94, 0.98)**	**<0.001**	**<0.001**
		After 7:48 PM	18,976	**1.11 (1.03, 1.20)**	**0.008**	

Proportion of non-meal energy intake	Crude model	Per 10% increase	34,974	**1.18 (1.14, 1.22)**	**<0.001**	**<0.001**
		Quartiles^d^	—	—	—	
		Q1 (<8.9%)	8746	Reference (1)	—	
		Q2 (8.9 to <18.25%)	8737	0.90 (0.76, 1.07)	0.236	
		Q3 (18.25 to <29.6%)	8750	**1.21 (1.02, 1.43)**	**0.03**	
		Q4 (≥29.6%)	8741	**1.61 (1.39, 1.87)**	**<0.001**	
	Adjusted model	Per 10% increase	34,974	**1.11 (1.08, 1.15)**	**<0.001**	0.249
		Quartiles	—	—	—	
		Q1 (<8.9%)	8746	Reference (1)	—	
		Q2 (8.9 to <18.25%)	8737	0.94 (0.78, 1.13)	0.492	
		Q3 (18.25 to <29.6%)	8750	**1.23 (1.03, 1.48)**	**0.024**	
		Q4 (≥29.6%)	8741	**1.44 (1.21, 1.70)**	**<0.001**	

*Note*: The bolded values are to highlight the results that are statistically significant (*p* < 0.05).

Abbreviations: CI, confidence interval; OR, odds ratio; Q, quartile.

^a^Detailed definitions of the exposures are provided in Table S5 in Supporting Information.

^b^Crude model were unadjusted. Adjusted model were adjusted for age, sex, race and ethnicity, education level, marital status, family income, body mass index, smoking status, drinking status, physical activity, comorbid condition, sleep duration, total energy intakes, Healthy Eating Index, and dietary recall day of the week.

^c^To examine the relationship between exposure and depressive symptoms, we conducted segmented logistic regression for exposure levels below and above the cut point. Participants with an exposure level equal to the cut point were excluded from the analysis, which may result in differences in the number of participants between the crude and adjusted models.

^d^The proportion of non-meal energy intake was categorized into quartiles to analyze its association with depressive symptoms.

**Table 3:** Association between meal energy intake behaviors and depressive symptoms.

Exposure^a^	Model^b^	Cut points^c^	No.^c^	OR (95% CI)	*p*-value	*p* for nonlinearity
Proportion of breakfast energy intake	Crude model	<18%	16,980	**0.66 (0.59, 0.75)**	**<0.001**	**<0.001**
		>18%	17,994	**1.12 (1.04, 1.20)**	**0.001**	
	Adjusted model	<20%	19,238	**0.85 (0.75, 0.96)**	**0.01**	**0.002**
		>20%	15,734	1.03 (0.95, 1.12)	0.462	

Breakfast time	Crude model	Before 7:06 AM	6480	0.88 (0.76, 1.01)	0.075	**<0.001**
		After 7:06 AM	25,668	**1.15 (1.10, 1.21)**	**<0.001**	
	Adjusted model	Before 7:54 AM	11,736	0.97 (0.85, 1.10)	0.612	0.077
		After 7:54 AM	20,412	**1.09 (1.03, 1.15)**	**0.003**	

Proportion of lunch energy intake	Crude model	<33%	25,267	**0.77 (0.73, 0.82)**	**<0.001**	**<0.001**
		>33%	9706	**1.12 (1.03, 1.23)**	**0.012**	
	Adjusted model	<37%	27,933	**0.91 (0.86, 0.95)**	**<0.001**	0.06
		>37%	7041	0.97 (0.86, 1.09)	0.576	

Lunch time	Crude model	Before 12:42 PM	15,183	**0.91 (0.86, 0.95)**	**<0.001**	**<0.001**
		After 12:42 PM	15,572	**1.09 (1.03, 1.14)**	**0.002**	
	Adjusted model	Before 12:42 PM	15,183	0.94 (0.88, 1.01)	0.091	**0.002**
		After 12:42 PM	15,572	1.04 (0.99, 1.10)	0.147	

Proportion of dinner energy intake	Crude model	<35%	17,953	**0.86 (0.80, 0.92)**	**<0.001**	**<0.001**
		>35%	17,019	**1.12 (1.05, 1.19)**	**<0.001**	
	Adjusted model	<39%	21,538	0.95 (0.89, 1.01)	0.113	0.762
		>39%	13,436	1.01 (0.93, 1.09)	0.901	

Dinner time	Crude model	Before 6:42 PM	17,558	**0.94 (0.91, 0.98)**	**0.003**	**<0.001**
		After 6:42 PM	16,066	1.07 (0.98, 1.17)	0.111	
	Adjusted model	Before 5:48 PM	9505	0.96 (0.91, 1.01)	0.158	**0.034**
		After 5:48 PM	24,119	1.04 (0.96, 1.12)	0.345	

*Note*: The bolded values are to highlight the results that are statistically significant (*p* < 0.05).

Abbreviations: CI, confidence interval; OR, odds ratio.

^a^Detailed definitions of the exposures are provided in Table S6 in Supporting Information.

^b^Crude model were unadjusted. Adjusted model were adjusted for age, sex, race and ethnicity, education level, marital status, family income, body mass index, smoking status, drinking status, physical activity, comorbid condition, sleep duration, total energy intakes, Healthy Eating Index, and dietary recall day of the week.

^c^To examine the relationship between exposure and depressive symptoms, we conducted segmented logistic regression for exposure levels below and above the cut point. Participants with an exposure level equal to the cut point were excluded from the analysis, which may result in differences in the number of participants between the crude and adjusted models.

## Data Availability

The data used in this study are available on the National Health and Nutrition Examination Survey website: https://www.cdc.gov/nchs/nhanes/index.htm

## Supporting Information

Supporting Information Figure S1. Flowchart for inclusion of study participants. Table S1. Characteristics of the included and excluded participants. Table S2. Univariate analysis of the association between depression with covariates. Table S3. Missing covariates. Table S4. 24-h energy intake in participants with and without depressive symptoms. Table S5. Definitions of exposures and related literature. Table S6. Association between energy intake and depressive symptoms at different time periods. Table S7. Characteristics of participants with different time of starting daily energy intake. Table S8. Characteristics of participants with different time of last daily energy intake. Table S9. Characteristics of participants with different proportion of non-meal energy intake. Table S10. Characteristics of participants with different proportion of breakfast energy intake. Table S11. Characteristics of participants with different breakfast time. Table S12. Characteristics of participants with different proportion of lunch energy intake. Table S13. Association between energy intake and depressive symptoms at different time periods. Table S14. Association between meals and depressive symptoms.
