# Supplementary material for: Association of 24-h Energy Intake Behavior With Depressive Symptoms: Findings From the National Health and Nutrition Examination Survey
Source: Depress Anxiety. 2025 Apr 15;2025:5544651. doi: 10.1155/da/5544651 (PMC12014258; doi:10.1155/da/5544651)
Supplement: Supporting Information — Figure S1. Flowchart for inclusion of study participants. Table S1. Characteristics of the included and excluded participants. Table S2. Univariate analysis of the association between depression with covariates. Table S3. Missing covariates. Table S4. 24-h energy intake in participants with and without depressive symptoms. Table S5. Definitions of exposures and related literature. Table S6. Association between energy intake and depressive symptoms at different time periods. Table S7. Characteristics of participants with different time of starting daily energy intake. Table S8. Characteristics of participants with different time of last daily energy intake. Table S9. Characteristics of participants with different proportion of non-meal energy intake. Table S10. Characteristics of participants with different proportion of breakfast energy intake. Table S11. Characteristics of participants with different breakfast time. Table S12. Characteristics of participants with different proportion of lunch energy intake. Table S13. Association between energy intake and depressive symptoms at different time periods. Table S14. Association between meals and depressive symptoms. [file 5544651.f1.docx]

**Supplementary Material**

**Supplementary Table 1. Detailed definition of covariates**

| Covariate | | Detailed definition | |
| --- | --- | --- | --- |
| Sex | Male | | |
|  | Female | | |
| Age | 18 to <40 years | | |
|  | 40 to <60 years | | |
|  | >=60 years | | |
| Race/ethnicity | Non-Hispanic white | | |
|  | Non-Hispanic black | | |
|  | Mexican American | | |
|  | Other Hispanic | | |
|  | Other race/multiple races | | |
| Education level | Less than high school | | |
|  | Completed high school | | |
|  | Beyond high school | | |
| Occupational status | Working at a job or business | | |
|  | With a job or business but not at work | | |
|  | Looking for work | | |
|  | Not working | | |
| Marital status | Married/Living with partner | | |
|  | Widowed/Divorced/Separated/Never married | | |
| Family income | Low income | | Family income was classified into three categories based on the family poverty income ratio, as employed by US government agencies^1^ to provide NHANES food and health data: low income (≤1.3), medium income (>1.3 to 3.5), and high income (>3.5). |
|  | Medium income | |  |
|  | High income | |  |
| Alcohol status | Never drinking | | The categories for alcohol status were: never (less than 12 drinks in a lifetime), former (more than 12 drinks in a single year and no drink the previous year, or more than 12 drinks in a lifetime), light/moderate drinker (less than 1 drink per day on average for women or less than 2 drinks per day on average for men), and heavy drinker (more than 1 drink per day on average for women or more than 2 drinks per day on average for men). |
|  | Former drinker | |  |
|  | Current light/moderate drinker | |  |
|  | Current heavier drinker | |  |
| Smoking status | Never smoking | | Smoked <100 cigarettes. |
|  | Former smoker | | Not currently smoking but smoked ≥100 cigarettes. |
|  | Current smoker | | ≥100 cigarettes and currently smoking every day or some days. |
| Physical activity | Inactive | | Reporting no moderate activity or vigorous activity. |
|  | Moderate | | Moderate physical activity (e.g., brisk walking, swimming, bicycling at a regular pace) reported. |
|  | Vigorous | | High-intensity activities, fitness and sports such as running or basketball reported. |
|  | Both moderate and vigorous | | Both moderate and vigorous activity reported. |
| BMI | <18.5 kg/m2 | | Weight (kg) divided by height (m) squared. |
|  | 18.5 to <25.0 kg/m2 | |  |
|  | 25.0 to <30.0 kg/m2 | |  |
|  | ≥30.0 kg/m2 | |  |
| Sleep duration | <6 h | | Sleep duration was obtained through the question: "How much sleep do you/does the sample person usually get at night on weekdays or workdays?" |
|  | 6 to 8 h | |  |
|  | >8 h | |  |
| Comorbid condition | Any participant who reported having at least one of the following medical problems—diabetes, renal failure, kidney stones, heart failure, stroke, hepatopath, rheumatoid arthritis, and cancer—was considered to have comorbid conditions. | | |
| Total energy intakes | Energy intake was assessed through 24-hour dietary recall interviews. Participants reported all foods and beverages consumed, and energy intake was estimated using the United States Department of Agriculture’s Food and Nutrient Database. The average of two 24-hour recalls served as the basis for total energy intake. If only one 24-hour recall was conducted, the whole energy consumption for that day represented the total energy intake. | | |
| HEI | The Healthy Eating Index (HEI) measures diet quality based on alignment with the Dietary Guidelines for Americans^2^. Scores range from 0 to 100, with higher scores indicating healthier diets. The total number of nutrients ingested in the twenty-four hours leading up to the in-person interview was used to compute HEI. | | |
| First dietary recall day of the week | The first dietary recall in NHANES is conducted in person at the Mobile Examination Center using the Automated Multiple-Pass Method. Participants report all foods and beverages consumed in the past 24 hours, including details on portion size, preparation, and timing. Trained interviewers guide the process to ensure accuracy and completeness. Some recalls were conducted on weekdays, while others were conducted on weekends. | | |
| Second dietary recall day of the week | The second dietary recall in NHANES is conducted by phone 3 to 10 days after the first recall, using the same Automated Multiple-Pass Method. Some recalls were conducted on weekdays, while others were conducted on weekends. | | |


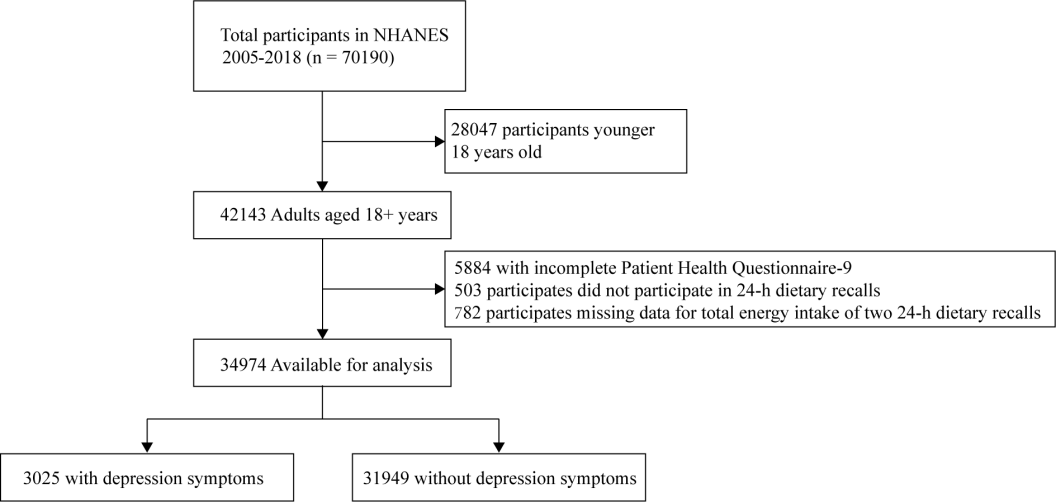


**Supplementary Figure 1. Flowchart for inclusion of study participants**

**Supplementary Table 2. Characteristics of the included and excluded participants**

| Characteristic | Excluded | Included | *p*-value |
| --- | --- | --- | --- |
|  |  |  |  |
|  |  |  |  |
| Sample size | 7169 | 34974 |  |
| Male, n (%) | 3249 (45.3) | 17209 (49.2) | <0.001 |
| Age, n (%) |  |  | 0.001 |
| 18 to <40 years | 2659 (37.1) | 13202 (37.7) |  |
| 40 to <60 years | 2090 (29.2) | 10712 (30.6) |  |
| >=60 years | 2420 (33.8) | 11060 (31.6) |  |
| Educational level, n (%) |  |  | <0.001 |
| Less than high school | 2363 (33.1) | 8610 (24.6) |  |
| Completed high school | 1591 (22.3) | 8401 (24.0) |  |
| Beyond high school | 3175 (44.5) | 17940 (51.3) |  |
| Race and ethnicity, n (%) |  |  | <0.001 |
| Non-Hispanic White | 2348 (32.8) | 14846 (42.4) |  |
| Non-Hispanic Black | 1610 (22.5) | 7643 (21.9) |  |
| Mexican American | 758 (10.6) | 3283 (9.4) |  |
| Other Hispanic | 1145 (16.0) | 5657 (16.2) |  |
| Other race/multiple races | 1308 (18.2) | 3545 (10.1) |  |
| BMI, n (%) |  |  | <0.001 |
| <18.5 kg/m2 | 141 (2.7) | 603 (1.7) | <0.001 |
| 18.5 to <25.0 kg/m^2^ | 1704 (32.6) | 9699 (28.0) |  |
| 25.0 to <30.0 kg/m^2^ | 1652 (31.6) | 11287 (32.6) |  |
| ≥30.0 kg/m^2^ | 1731 (33.1) | 13058 (37.7) |  |
| Marital status, n (%) |  |  | <0.001 |
| Married/Living with partner | 3815 (55.3) | 19728 (59.1) |  |
| Never married/Widowed /Divorced/Separated | 3087 (44.7) | 13669 (40.9) |  |
| Drinking status, n (%) |  |  | <0.001 |
| Never drinking | 456 (28.3) | 4899 (14.9) |  |
| Former drinker | 303 (18.8) | 5300 (16.1) |  |
| Current light/moderate drinker | 607 (37.7) | 15974 (48.5) |  |
| Current heavier drinker | 246 (15.3) | 6757 (20.5) |  |
| Smoking status, n (%) |  |  | <0.001 |
| Never smoking | 4151 (59.6) | 18725 (55.6) |  |
| Former smoker | 1434 (20.6) | 8093 (24.0) |  |
| Current smoker | 1375 (19.8) | 6843 (20.3) |  |
| Physical activity, n (%) |  |  |  |
| Inactive | 3718 (58.9) | 15568 (51.4) | <0.001 |
| Moderate | 1337 (21.2) | 7648 (25.3) |  |
| Vigorous | 459 (7.3) | 2450 (8.1) |  |
| Both moderate and vigorous | 795 (12.6) | 4603 (15.2) |  |
| ^a^Family income, n (%) |  |  |  |
| Low income | 2303 (38.6) | 10284 (32.0) | <0.001 |
| Medium income | 2183 (36.5) | 12093 (37.7) |  |
| High income | 1487 (24.9) | 9721 (30.3) |  |
| Occupational status, n (%) |  |  | <0.001 |
| Working at a job or business | 3446 (48.1) | 18775 (53.7) |  |
| With a job or business but not at work | 98 (1.4) | 729 (2.1) |  |
| Looking for work | 185 (2.6) | 1298 (3.7) |  |
| Not working | 3435 (47.9) | 14153 (40.5) |  |
| Comorbid condition, n (%) |  |  | <0.001 |
| No | 4196 (59.5) | 19623 (56.3) |  |
| Yes | 2855 (40.5) | 15205 (43.7) |  |
| Sleep duration, n (%) |  |  | <0.001 |
| <6 h | 1002 (14.1) | 4688 (13.4) |  |
| 6 to 8 h | 4913 (69.0) | 25161 (72.1) |  |
| >8 h | 1209 (17.0) | 5033 (14.4) |  |
| Total energy intakes, n (%) |  |  | <0.001 |
| Quartile 1 (<1458 kcal) | 733 (29.2) | 8644 (24.7) |  |
| Quartile 2 (1458–1916.5 kcal) | 667 (26.6) | 8703 (24.9) |  |
| Quartile 3 (1917–2491 kcal) | 586 (23.4) | 8779 (25.1) |  |
| Quartile 4 (>2491 kcal) | 523 (20.8) | 8848 (25.3) |  |
| HEI, n (%) |  |  | 0.014 |
| Quartile 1 (<40.396) | 576 (23.0) | 8796 (25.2) |  |
| Quartile 2 (40.396–49.668) | 607 (24.2) | 8762 (25.1) |  |
| Quartile 3 (49.669–59.543) | 646 (25.7) | 8725 (24.9) |  |
| Quartile 4 (>59.543) | 680 (27.1) | 8691 (24.8) |  |
| First dietary recall time, n (%) |  |  | 0.045 |
| weekday | 1747 (63.4) | 21480 (61.4) |  |
| weekend | 1010 (36.6) | 13494 (38.6) |  |
| Second dietary recall time, n (%) |  |  | 0.846 |
| weekday | 1560 (79.2) | 24575 (79.4) |  |
| weekend | 410 (20.8) | 6377 (20.6) |  |

Abbreviations: BMI, body mass index; HEI, Healthy Eating Index.

a. Family income was categorized into three levels based on the family poverty income ratio (PIR): low-income (PIR ≤ 1.3), medium-income (1.3 < PIR ≤ 3.5), and high-income (PIR > 3.5).

**Supplementary Table 3. Univariate analysis of the association between depressive symptoms with covariates**

|  | N (percentage%) | OR (95% CI) | *P* value |
| --- | --- | --- | --- |
| Sex |  |  |  |
| Female | 17765 (50.79%) | Reference |  |
| Male | 17209 (49.21%) | 0.60 (0.53,0.68) | <0.001 |
| Age groups |  |  |  |
| 18 to <40 years | 13202 (37.75%) | Reference |  |
| 40 to <60 years | 10712 (30.63%) | 1.25 (1.11,1.41) | <0.001 |
| >=60 years | 11060 (31.62%) | 0.80 (0.69,0.93) | 0.004 |
| Race and ethnicity |  |  |  |
| Non-Hispanic White | 14846 (42.45%) | Reference |  |
| Non-Hispanic Black | 7643 (21.85%) | 1.34 (1.17,1.53) | <0.001 |
| Mexican American | 3283 (9.39%) | 1.65 (1.36,2.00) | <0.001 |
| Other Hispanic | 5657 (16.17%) | 1.08 (0.89,1.32) | 0.44 |
| Other race/multiple races | 3545 (10.14%) | 1.04 (0.82,1.32) | 0.75 |
| Educational level |  |  |  |
| Less than high school | 8610 (24.63%) | Reference |  |
| Completed high school | 8401 (24.04%) | 0.71 (0.62,0.81) | <0.001 |
| Beyond high school | 17940 (51.33%) | 0.46 (0.40,0.53) | <0.001 |
| Occupational status |  |  |  |
| Working at a job or business | 18775 (53.71%) | Reference |  |
| With a job or business but not at work | 729 (2.09%) | 0.71 (0.49,1.03) | 0.07 |
| Looking for work | 1298 (3.71%) | 2.28 (1.78,2.91) | <0.001 |
| Not working | 14153 (40.49%) | 2.70 (2.42,3.00) | <0.001 |
| ^a^Family income |  |  |  |
| Low income | 10284 (32.04%) | Reference |  |
| Medium income | 12093 (37.68%) | 0.49 (0.43,0.56) | <0.001 |
| High income | 9721 (30.29%) | 0.23 (0.20,0.27) | <0.001 |
| Marital status |  |  |  |
| Never married/Widowed /Divorced/Separated | 13669 (40.93%) | Reference |  |
| Married/Living with partner | 19728 (59.07%) | 0.51 (0.46,0.56) | <0.001 |
| Drinking status |  |  |  |
| Never drinking | 4899 (14.88%) | Reference |  |
| Former drinker | 5300 (16.09%) | 1.77(1.45,2.18) | <0.001 |
| Current light/moderate drinker | 15974 (48.51%) | 0.99(0.84,1.16) | 0.9 |
| Current heavier drinker | 6757 (20.52%) | 1.47(1.22,1.76) | <0.001 |
| Smoking status |  |  |  |
| Never smoking | 18725 (55.63%) | Reference |  |
| Former smoker | 8093 (24.04%) | 1.22 (1.04,1.44) | 0.02 |
| Current smoker | 6843 (20.33%) | 3.14 (2.75,3.60) | <0.001 |
| Physical activity |  |  |  |
| Inactive | 15568 (51.43%) | Reference |  |
| Moderate | 7648 (25.27%) | 0.52 (0.44,0.61) | <0.001 |
| Vigorous | 2450 (8.09%) | 0.31 (0.23,0.42) | <0.001 |
| Both moderate and vigorous | 4603 (15.21%) | 0.26 (0.20,0.34) | <0.001 |
| BMI category |  |  |  |
| <18.5 kg/m2 | 603 (1.74%) | 1.54 (1.07,2.21) | 0.02 |
| 18.5 to <25.0 kg/m^2^ | 9699 (27.99%) | Reference |  |
| 25.0 to <30.0 kg/m^2^ | 11287 (32.58%) | 0.92 (0.78,1.07) | 0.27 |
| ≥30.0 kg/m^2^ | 13058 (37.69%) | 1.54 (1.32,1.79) | <0.0001 |
| Comorbid condition |  |  |  |
| No | 19623 (56.34%) | Reference |  |
| Yes | 15205 (43.66%) | 1.88 (1.72,2.04) | <0.001 |
| Sleep duration |  |  |  |
| <6 h | 4688 (13.44%) | 3.62 (3.14,4.17) | <0.001 |
| 6 to 8 h | 25161 (72.13%) | Reference |  |
| >8 h | 5033 (14.43%) | 1.71 (1.43,2.05) | <0.001 |
| Total energy intakes |  |  |  |
| Quartile 1 (<1458 kcal) | 8744 (25%) | Reference |  |
| Quartile 2 (1458–1916.5 kcal) | 8745 (25%) | 0.68 (0.59,0.79) | <0.001 |
| Quartile 3 (1917–2491 kcal) | 8748 (25.01%) | 0.64 (0.56,0.74) | <0.001 |
| Quartile 4 (>2491 kcal) | 8737 (24.98%) | 0.59 (0.52,0.67) | <0.001 |
| HEI |  |  |  |
| Quartile 1 (<40.396) | 8744 (25%) | Reference |  |
| Quartile 2 (40.396–49.668) | 8742 (25%) | 0.80 (0.68,0.94) | 0.01 |
| Quartile 3 (49.669–59.543) | 8745 (25%) | 0.69 (0.60,0.80) | <0.001 |
| Quartile 4 (>59.543) | 8743 (25%) | 0.46 (0.39,0.53) | <0.001 |
| First dietary recall time |  |  |  |
| weekday | 21480 (61.42%) | Reference |  |
| weekend | 13494 (38.58%) | 0.87 (0.78,0.98) | 0.02 |
| Second dietary recall time |  |  |  |
| weekday | 24575 (79.4%) | Reference |  |
| weekend | 6377 (20.6%) | 0.96 (0.84,1.08) | 0.49 |
| ^b^Duration of recreational physical activity |  |  |  |
| Less than 60 minutes | 16464 (47.1%) | Reference |  |
| 60–<150 minutes | 3479 (10.0%) | 0.85 (0.58,1.26) | <0.0001 |
| More than 150 minutes | 10294 (29.4%) | 0.53 (0.37,0.76) | <0.0001 |
| ^c^Work hours |  |  |  |
| 0 | 16180 (46.3%) | Reference |  |
| 1-34 | 4875 (13.9%) | 0.52 (0.44,0.63) | <0.0001 |
| 35-40 | 7321 (20.9%) | 0.36 (0.31,0.42) | <0.0001 |
| 41-55 | 4452 (12.7%) | 0.32 (0.25,0.40) | <0.0001 |
| More than 55 | 2109 (6.0%) | 0.41 (0.30,0.55) | <0.0001 |

Abbreviations: BMI, body mass index; HEI, Healthy Eating Index.

a.Family income was categorized into three levels based on the family poverty income ratio (PIR): low-income (PIR ≤ 1.3), medium-income (1.3 < PIR ≤ 3.5), and high-income (PIR > 3.5).

b.The duration of recreational physical activity was obtained by asking participants the questions: "How much time do you spend doing moderate-intensity/vigorous-intensity sports, fitness, or recreational activities on a typical day?" and "In a typical week, on how many days do you do moderate-intensity/vigorous-intensity sports, fitness, or recreational activities?"

c.Work hours were obtained by asking the question: "How many hours did you work last week at all jobs or businesses?"

**Supplementary Table 4. Missing covariates**

| Variable | Number of participants (% missing) |
| --- | --- |
| Age | 0 (0%) |
| Sex | 0 (0%) |
| Race and ethnicity | 0 (0%) |
| Educational level | 23 (0.07%) |
| Occupational status | 19 (0.05%) |
| Family income | 2876 (8.22%) |
| Marital status | 1577 (4.51%) |
| Body mass index | 327 (0.93%) |
| Physical activity | 4705 (13.45%) |
| Smoking status | 1313 (3.75%) |
| Drinking status | 2044 (5.84%) |
| Sleep duration | 92 (0.26%) |
| Comorbid condition | 146 (0.42%) |
| Healthy Eating Index | 0 (0%) |
| Diurnal total energy intake | 0 (0%) |
| First dietary recall time | 0 (0%) |
| Second dietary recall time | 4022 (11.50%) |

**Supplementary Table 5. 24-hour energy intake in participants with and without depressive symptoms**

| Time Periods^a^ | Participants without depressive symptoms | Participants with depressive symptoms | *P* value |
| --- | --- | --- | --- |
| 0:00-1:00 | **0.26 (0.22, 0.30)** | **0.43 (0.29, 0.56)** | **0.018** |
| 1:00-2:00 | **0.18 (0.15, 0.21)** | **0.35 (0.18, 0.53)** | **0.049** |
| 2:00-3:00 | 0.17 (0.14, 0.19) | 0.26 (0.15, 0.37) | 0.091 |
| 3:00-4:00 | 0.14 (0.12, 0.17) | 0.22 (0.14, 0.30) | 0.073 |
| 4:00-5:00 | 0.24 (0.20, 0.27) | 0.36 (0.22, 0.49) | 0.103 |
| 5:00-6:00 | 0.67 (0.60, 0.74) | 0.58 (0.44, 0.73) | 0.223 |
| 6:00-7:00 | 1.75 (1.66, 1.84) | 1.45 (1.11, 1.79) | 0.085 |
| 7:00-8:00 | **3.69 (3.56, 3.83)** | **2.89 (2.53, 3.25)** | **< 0.001** |
| 8:00-9:00 | **4.83 (4.67, 4.98)** | **4.37 (4.00, 4.74)** | **0.024** |
| 9:00-10:00 | 4.38 (4.23, 4.53) | 4.62 (4.22, 5.01) | 0.265 |
| 10:00-11:00 | **3.87 (3.76, 3.99)** | **4.50 (4.08, 4.92)** | **0.003** |
| 11:00-12:00 | 5.14 (4.98, 5.31) | 5.27 (4.75, 5.79) | 0.639 |
| 12:00-13:00 | **10.61 (10.35, 10.86)** | **8.66 (8.05, 9.26)** | **< 0.001** |
| 13:00-14:00 | 6.99 (6.79, 7.18) | 7.08 (6.51, 7.65) | 0.76 |
| 14:00-15:00 | 4.68 (4.53, 4.83) | 4.77 (4.30, 5.24) | 0.714 |
| 15:00-16:00 | **4.12 (3.98, 4.25)** | **4.93 (4.50, 5.36)** | **< 0.001** |
| 16:00-17:00 | **4.13 (3.97, 4.30)** | **4.78 (4.31, 5.25)** | **0.007** |
| 17:00-18:00 | 7.39 (7.12, 7.65) | 7.42 (6.83, 8.01) | 0.91 |
| 18:00-19:00 | **11.59 (11.30, 11.88)** | **10.30 (9.40, 11.20)** | **0.009** |
| 19:00-20:00 | 10.16 (9.89, 10.43) | 9.59 (8.85, 10.34) | 0.159 |
| 20:00-21:00 | 6.94 (6.74, 7.13) | 7.15 (6.62, 7.67) | 0.44 |
| 21:00-22:00 | **4.25 (4.10, 4.41)** | **4.91 (4.41, 5.41)** | **0.013** |
| 22:00-23:00 | **2.57 (2.45, 2.68)** | **3.10 (2.75, 3.46)** | **0.006** |
| 23:00-0:00 | **1.22 (1.12, 1.32)** | **1.86 (1.56, 2.15)** | **< 0.001** |

^a^We divided the time into hourly periods, containing the beginning of the hour to the end of the hour, for example, 0:00-1:00 means from 0:00 to before 1:00.

**Supplementary Table 6. Definitions of exposures and related literature**

| Exposure | Related literature | Definition^a^ |
| --- | --- | --- |
| Proportion of non-meal energy intake | Paans et al., 2019^3^  Sangouni et al., 2022^4^  Sousa et al., 2019^5^ | Dietary energy intake was estimated using the United States Department of Agriculture’s Food and Nutrient Database for Dietary Studies. Each NHANES recall collects information on a name and clock time for each eating event reported. All recalled food or beverage items reported at one clock time are given the same eating event name. A total of 1,039,622 eating records were recorded in the two 24-hour dietary recalls, of which 27 eating records did not report the meal name. Energy consumed from “snack,” “drink,” “extended consumption,” “merienda” (afternoon snack or tea), “entre comida” (between meals), “tentempie” (snack or bite to eat), “botana” (snack), “bebida” (drink), and “bocadillo” (snack or bite) was defined as non-meal energy intake. Energy consumed from “breakfast,” “brunch,” “lunch,” “dinner,” “supper,” “desayuno” (breakfast), “almuerzo” (breakfast), “comida” (lunch), and “cena” (dinner) was defined as meal energy intake. The proportion of non-meal energy intake was defined as the proportion of non-meal energy intake to the total energy intake in the 24-hour dietary recall. For participants who obtained data from two recalls, their proportion of non-meal energy intake was defined by the mean of two recalls (n=30,814). For participants who obtained data from only one recall, their proportion of non-meal energy intake was defined as the proportion of non-meal energy intake at that recall (n=4,160). A total of 34,974 participants were included in the analysis of the relationship between the proportion of non-meal energy intake and depressive symptoms. |
| Time of starting daily energy intake | NA | In the NHANES data, the times for food intake are recorded as the 24 hours before the interview (i.e., from midnight to midnight) as outlined in the dietary recall protocol. The time of the collected food intake event is the time when the food intake event started. Since the 24-hour recall captures intake from the last 24-hour, there is a possibility that recall times capture energy intake from the previous night for participants who ate after midnight. Therefore, we set the start of a behavioral day to 5:00 AM^6^. The first and last mealtimes were recorded as meals after 5:00 AM and before 4:59 AM, respectively. Thus, energy consumed between midnight to 4:59 AM was considered late-night energy consuming. For participants who participated in two dietary recalls, their start/end times were defined as the average of the 2 recalls (n=30,814). For participants who participated in only one dietary recall (n=4160), their start/end times were defined by that recall. |
| Time of last daily energy intake | NA |  |
| Breakfast time | Wilson et al., 2020^7^ | We defined “breakfast,” “desayuno” (Spanish), and “almuerzo” (Spanish) as breakfast^8,9^. For participants who reported breakfast time in both dietary recalls, their breakfast time was defined as the average of two days of breakfast time (n=23,656), and for participants who reported breakfast time only once, their breakfast time was defined as the time they reported (n=8,492). In total, 32,148 participants were included in the analysis of the relationship between breakfast time and depressive symptoms. |
| Lunch time | NA | We defined “lunch,” “brunch,” and “comida” (Spanish)” as lunch^8,10^. For participants who reported lunch time in both dietary recalls, their lunch time was defined as the average of two days of lunch time (n=19,516), and for participants who reported lunch time only once, their lunch time was defined as the time they reported (n=11,239). A total of 30,755 participants were included in the analysis of the relationship between lunch time and depressive symptoms. |
| Dinner time | Suzuki et al., 2016^11^ | We defined “dinner,” “supper,” and “cena” (Spanish) as dinner^8,10^. For participants who reported dinner time in both dietary recalls, their dinner time was defined as the average of two days of dinner time (n=25,904), and for participants who reported dinner time only once, their dinner time was defined as the time they reported (n=7,720). In total, 33,624 participants were included in the analysis of the relationship between dinner time and depressive symptoms. |
| Proportion of breakfast energy intake | NA | The proportion of breakfast energy intake was defined as the proportion of breakfast energy intake in the total energy intake of the 24-hour dietary recall. For participants with data from both dietary recalls, their proportion of breakfast energy intake was defined as the average of the two recalls (n=30,814). For participants who obtained data from only one 24-hour dietary recall, their breakfast energy intake was defined by that recall (n=4,160). A total of 34,974 participants were included in the analysis of the relationship between the proportion of breakfast energy intake and depressive symptoms. |
| Proportion of lunch energy intake | NA | The proportion of lunch energy intake was defined as the proportion of lunch energy intake in the total energy intake of the 24-hour dietary recall. For participants with data from both dietary recalls, their proportion of lunch energy intake was defined as the average of the two recalls (n=30,814). For participants who obtained data from only one 24-hour dietary recall, their lunch energy intake was defined by that recall (n=4,160). In total, 34,974 participants were included in the analysis of the relationship between the proportion of lunch energy intake and depressive symptoms. |
| Proportion of dinner energy intake | NA | The proportion of dinner energy intake was defined as the proportion of dinner energy intake in the total energy intake of the 24-hour dietary recall. For participants with data from both dietary recalls, their proportion of dinner energy intake was defined as the average of the two recalls (n=30,814). For participants who obtained data from only one 24-hour dietary recall, their dinner energy intake was defined by that recall (n=4,160). A total of 34,974 participants were included in the analysis of the relationship between the proportion of dinner energy intake and depressive symptoms. |
| Eating breakfast | Zahedi et al., 2022^12^ | Skipping breakfast at both dietary recalls: two dietary recalls were attended, and skipping breakfast was reported in both recalls |
|  |  | Participated in one dietary recall and reported skipping breakfast: only one dietary recall (in-person interview or telephone interview) was attended, and skipping breakfast was reported |
|  |  | Participated in both dietary recall and reported eating breakfast only once: participated in two dietary recalls, and reported eating breakfast on one recall and skipping breakfast on the other recall |
|  |  | Participated in one dietary recall and reported eating breakfast: only one dietary recall (in-person interview or telephone interview) was attended, and eating breakfast was reported |
|  |  | Both dietary recalls reported eating breakfast: two dietary recalls were attended, and eating breakfast was reported in both recalls |
| Eating lunch | Fulkerson et al., 2004^13^  Yun et al., 2021^14^ | See “Eating breakfast” for definition |
| Eating dinner | Kwak and Kim, 2018^15^  Tajik et al., 2016^16^ | See “Eating breakfast” for definition |

NA, not applicable; NHANES, National Health and Nutrition Survey.

**Supplementary Table 7. Characteristics of participants with different time of starting daily energy intake**

| Characteristic | Time of Starting Daily Energy Intake | | |  |
| --- | --- | --- | --- | --- |
|  | Before 7:00 AM | Between 7:00 and before 9:00 AM | 9:00 AM or later | *P* value |
|  | (n = 5479) | (n = 16563) | (n = 12932) |  |
| Physical activity |  |  |  | < 0.0001 |
| Inactive | 37.14 (34.55,39.74) | 37.40 (35.71,39.09) | 42.65 (40.79,44.52) |  |
| Moderate | 27.01 (24.78,29.24) | 25.90 (24.65,27.14) | 19.90 (18.69,21.11) |  |
| Vigorous | 6.33 (5.38,7.28) | 6.76 (6.09,7.43) | 8.58 (7.79,9.38) |  |
| Both moderate and vigorous | 15.40 (13.66,17.14) | 16.74 (15.46,18.03) | 15.85 (14.50,17.21) |  |
| Occupational status |  |  |  | < 0.0001 |
| Working at a job or business | 67.96 (66.03,69.90) | 58.10 (56.67,59.54) | 55.90 (54.52,57.27) |  |
| With a job or business but not at work | 2.35 (1.71,2.99) | 2.41 (2.02,2.81) | 2.68 (2.18,3.19) |  |
| Looking for work | 1.52 (1.07,1.97) | 2.14 (1.85,2.43) | 5.66 (5.07,6.25) |  |
| Not working | 28.15 (26.22,30.07) | 37.26 (35.82,38.70) | 35.73 (34.40,37.06) |  |
| Family income |  |  |  | < 0.0001 |
| Low income | 14.42 (12.96,15.87) | 17.29 (16.11,18.48) | 29.20 (27.38,31.02) |  |
| Medium income | 30.13 (28.07,32.20) | 32.72 (31.15,34.29) | 34.37 (32.49,36.24) |  |
| High income | 48.72 (46.00,51.43) | 43.69 (41.57,45.81) | 29.36 (27.29,31.43) |  |
| BMI |  |  |  | < 0.0001 |
| <18.5 kg/m2 | 1.44 (0.95,1.92) | 1.22 (0.99,1.45) | 2.36 (1.98,2.75) |  |
| 18.5 to <25.0 kg/m2 | 26.83 (24.90,28.76) | 27.85 (26.58,29.12) | 31.48 (30.11,32.85) |  |
| 25.0 to <30.0 kg/m2 | 36.01 (33.92,38.10) | 34.34 (33.28,35.39) | 28.06 (26.76,29.37) |  |
| ≥30.0 kg/m2 | 35.73 (33.68,37.78) | 36.59 (35.30,37.88) | 38.10 (36.52,39.67) |  |
| Total energy intakes |  |  |  | < 0.0001 |
| Quartile 1 (<1458 kcal) | 19.46 (17.84,21.08) | 21.10 (20.05,22.15) | 25.01 (23.94,26.08) |  |
| Quartile 2 (1458–1916.5 kcal) | 23.98 (22.36,25.60) | 26.26 (25.33,27.18) | 24.16 (23.07,25.25) |  |
| Quartile 3 (1917–2491 kcal) | 27.30 (25.53,29.08) | 26.78 (25.83,27.74) | 24.42 (23.28,25.56) |  |
| Quartile 4 (>2491 kcal) | 29.26 (27.50,31.02) | 25.86 (24.75,26.98) | 26.41 (25.31,27.51) |  |
| Duration of recreational physical activity^a^ |  |  |  | < 0.0001 |
| Less than 60 minutes | 46.24 (43.66,48.83) | 46.73 (44.94,48.52) | 51.70 (49.68,53.71) |  |
| 60–<150 minutes | 13.17 (11.65,14.70) | 13.42 (12.53,14.31) | 11.31 (10.20,12.42) |  |
| More than 150 minutes | 40.59 (38.01,43.16) | 39.86 (38.05,41.66) | 36.99 (35.33,38.66) |  |
| HEI |  |  |  | < 0.0001 |
| Quartile 1 (<40.396) | 21.35 (19.56,23.14) | 20.83 (19.67,21.99) | 31.66 (30.22,33.09) |  |
| Quartile 2 (40.396–49.668) | 23.29 (21.55,25.03) | 23.77 (22.69,24.85) | 26.81 (25.78,27.84) |  |
| Quartile 3 (49.669–59.543) | 25.34 (23.69,26.98) | 25.53 (24.52,26.53) | 23.51 (22.24,24.79) |  |
| Quartile 4 (>59.543) | 30.02 (27.62,32.42) | 29.88 (28.39,31.37) | 18.02 (16.61,19.44) |  |
| Work hours^b^ |  |  |  | < 0.0001 |
| 0 | 32.04 (30.10,33.98) | 41.86 (40.42,43.30) | 44.10 (42.72,45.48) |  |
| 1-34 | 12.45 (11.13,13.77) | 14.59 (13.66,15.53) | 16.74 (15.69,17.79) |  |
| 35-40 | 23.42 (21.88,24.97) | 20.92 (19.98,21.87) | 20.65 (19.48,21.81) |  |
| 41-55 | 21.02 (19.26,22.79) | 16.32 (15.36,17.27) | 12.42 (11.47,13.37) |  |
| More than 55 | 11.07 (9.71,12.43) | 6.31 (5.73, 6.89) | 6.10 (5.49, 6.70) | < 0.0001 |

^a^The duration of recreational physical activity was obtained by asking participants the questions: "How much time do you spend doing moderate-intensity/vigorous-intensity sports, fitness, or recreational activities on a typical day?" and "In a typical week, on how many days do you do moderate-intensity/vigorous-intensity sports, fitness, or recreational activities?"

^b^Work hours were obtained by asking the question: "How many hours did you work last week at all jobs or businesses?"

| **Supplementary Table 8. Characteristics of participants with different time of last daily energy intake** | | | | |
| --- | --- | --- | --- | --- |
| Characteristic | Time of Last Daily Energy Intake | | |  |
|  | Before 7:00 PM | Between 7:00 and before 8:00 PM | 8:00 PM or later | *P* value |
|  | (n = 9263) | (n = 6858) | (n = 18853) |  |
| Physical activity |  |  |  | < 0.0001 |
| Inactive | 42.58 (40.72,44.43) | 37.48 (35.38,39.57) | 38.02 (36.16,39.87) |  |
| Moderate | 23.61 (21.74,25.48) | 25.86 (24.16,27.55) | 23.69 (22.53,24.85) |  |
| Vigorous | 6.62 (5.84,7.40) | 6.54 (5.78,7.30) | 7.89 (7.18,8.61) |  |
| Both moderate and vigorous | 13.55 (11.95,15.15) | 16.35 (14.53,18.18) | 17.44 (16.19,18.70) |  |
| Occupational status |  |  |  | < 0.0001 |
| Working at a job or business | 56.20 (54.38,58.02) | 58.27 (56.35,60.18) | 60.89 (59.60,62.19) |  |
| With a job or business but not at work | 2.05 (1.64,2.47) | 2.56 (1.88,3.23) | 2.68 (2.28,3.08) |  |
| Looking for work | 2.40 (1.99,2.81) | 2.77 (2.22,3.32) | 3.74 (3.35,4.13) |  |
| Not working | 39.33 (37.52,41.13) | 36.21 (34.34,38.08) | 32.67 (31.33,34.00) |  |
| Family income |  |  |  | < 0.0001 |
| Low income | 22.52 (20.96,24.07) | 17.17 (15.89,18.45) | 21.20 (19.79,22.60) |  |
| Medium income | 34.20 (32.53,35.88) | 32.40 (30.45,34.34) | 32.27 (30.81,33.74) |  |
| High income | 36.68 (34.76,38.60) | 43.72 (41.28,46.15) | 39.91 (37.92,41.91) |  |
| BMI |  |  |  | 0.002 |
| <18.5 kg/m2 | 1.56 (1.20,1.92) | 1.29 (0.90,1.68) | 1.80 (1.53,2.08) |  |
| 18.5 to <25.0 kg/m^2^ | 27.53 (26.04,29.02) | 27.29 (25.47,29.10) | 30.11 (28.92,31.31) |  |
| 25.0 to <30.0 kg/m^2^ | 32.61 (30.94,34.28) | 33.09 (31.41,34.78) | 32.33 (31.30,33.36) |  |
| ≥30.0 kg/m^2^ | 38.30 (36.64,39.95) | 38.33 (36.30,40.37) | 35.75 (34.42,37.09) |  |
| Total energy intakes |  |  |  | < 0.0001 |
| Quartile 1 (<1458 kcal) | 29.23 (27.88,30.58) | 24.13 (22.66,25.61) | 17.89 (17.12,18.66) |  |
| Quartile 2 (1458–1916.5 kcal) | 24.63 (23.28,25.98) | 28.29 (26.71,29.88) | 24.23 (23.36,25.10) |  |
| Quartile 3 (1917–2491 kcal) | 21.33 (20.13,22.53) | 25.86 (24.28,27.45) | 28.48 (27.60,29.37) |  |
| Quartile 4 (>2491 kcal) | 24.81 (23.44,26.18) | 21.71 (19.87,23.55) | 29.40 (28.48,30.31) |  |
| Duration of recreational physical activity |  |  |  | < 0.0001 |
| Less than 60 minutes | 52.34 (50.51,54.17) | 46.63 (44.32,48.95) | 46.96 (44.87,49.05) |  |
| 60–<150 minutes | 11.33 (10.15,12.51) | 13.97 (12.66,15.27) | 12.84 (11.93,13.74) |  |
| More than 150 minutes | 36.33 (34.54,38.11) | 39.40 (37.17,41.64) | 40.20 (38.30,42.10) |  |
| HEI |  |  |  | 0.01 |
| Quartile 1 (<40.396) | 25.94 (24.55,27.32) | 23.23 (21.61,24.86) | 24.31 (23.19,25.44) |  |
| Quartile 2 (40.396–49.668) | 25.82 (24.42,27.21) | 23.80 (22.32,25.29) | 24.48 (23.50,25.45) |  |
| Quartile 3 (49.669–59.543) | 24.25 (23.02,25.48) | 25.47 (23.83,27.10) | 24.86 (23.92,25.80) |  |
| Quartile 4 (>59.543) | 24.00 (22.29,25.71) | 27.50 (25.69,29.30) | 26.35 (24.81,27.88) |  |
| Work hours |  |  |  | < 0.001 |
| 0 | 43.81 (41.99,45.63) | 41.62 (39.71,43.53) | 39.11 (37.81,40.41) |  |
| 1-34 | 13.00 (11.76,14.24) | 14.37 (13.04,15.70) | 16.06 (15.14,16.97) |  |
| 35-40 | 20.91 (19.55,22.27) | 21.42 (19.83,23.01) | 21.41 (20.46,22.36) |  |
| 41-55 | 15.01 (13.71,16.31) | 16.23 (14.90,17.56) | 16.15 (15.29,17.01) |  |
| More than 55 | 7.27 (6.32,8.21) | 6.36 (5.40,7.32) | 7.28 (6.64,7.92) |  |

| **Supplementary Table 9. Characteristics of participants with different proportion of non-meal energy intake** | | | | |
| --- | --- | --- | --- | --- |
| Characteristic | Proportion of Non-meal Energy Intake | | |  |
|  | ≤12% | >12%-25.2% | >25.2% | *P* value |
|  | (n = 11621) | (n = 11673) | (n = 11680) |  |
| Physical activity |  |  |  | 0.02 |
| Inactive | 40.71 (38.96,42.46) | 37.75 (35.70,39.80) | 38.97 (36.99,40.95) |  |
| Moderate | 23.23 (21.82,24.63) | 24.75 (23.36,26.13) | 24.27 (22.89,25.65) |  |
| Vigorous | 7.28 (6.47,8.08) | 7.98 (7.22,8.73) | 6.63 (5.88,7.38) |  |
| Both moderate and vigorous | 15.23 (13.86,16.59) | 16.08 (14.64,17.52) | 17.22 (15.75,18.69) |  |
| Occupational status |  |  |  | 0.43 |
| Working at a job or business | 58.71 (57.13,60.29) | 59.09 (57.62,60.56) | 59.57 (57.85,61.30) |  |
| With a job or business but not at work | 2.48 (2.01,2.95) | 2.54 (2.05,3.03) | 2.46 (2.00,2.92) |  |
| Looking for work | 2.92 (2.39,3.46) | 3.09 (2.64,3.54) | 3.55 (3.09,4.00) |  |
| Not working | 35.82 (34.32,37.31) | 35.26 (33.77,36.75) | 34.34 (32.64,36.03) |  |
| Family income |  |  |  | < 0.001 |
| Low income | 20.74 (19.47,22.01) | 18.80 (17.46,20.14) | 22.62 (20.87,24.37) |  |
| Medium income | 33.25 (31.53,34.97) | 33.26 (31.80,34.73) | 31.94 (30.25,33.64) |  |
| High income | 38.97 (36.90,41.03) | 41.25 (39.23,43.26) | 39.23 (36.97,41.49) |  |
| BMI |  |  |  | < 0.0001 |
| <18.5 kg/m2 | 1.36 (1.05,1.66) | 1.44 (1.10,1.77) | 2.09 (1.71,2.46) |  |
| 18.5 to <25.0 kg/m^2^ | 26.76 (25.39,28.12) | 28.00 (26.54,29.46) | 31.64 (30.20,33.07) |  |
| 25.0 to <30.0 kg/m^2^ | 31.94 (30.43,33.45) | 32.85 (31.61,34.09) | 32.83 (31.44,34.21) |  |
| ≥30.0 kg/m^2^ | 39.95 (38.47,41.42) | 37.71 (36.19,39.24) | 33.45 (32.07,34.83) |  |
| Total energy intakes |  |  |  | < 0.0001 |
| Quartile 1 (<1458 kcal) | 28.16 (26.97,29.35) | 20.61 (19.54,21.68) | 18.08 (17.01,19.16) |  |
| Quartile 2 (1458–1916.5 kcal) | 27.15 (26.10,28.19) | 25.81 (24.58,27.05) | 22.70 (21.65,23.74) |  |
| Quartile 3 (1917–2491 kcal) | 24.41 (23.19,25.64) | 26.50 (25.14,27.86) | 27.21 (26.08,28.34) |  |
| Quartile 4 (>2491 kcal) | 20.28 (19.09,21.47) | 27.08 (25.83,28.33) | 32.01 (30.79,33.23) |  |
| Duration of recreational physical activity |  |  |  | 0.003 |
| Less than 60 minutes | 50.44 (48.62,52.27) | 46.88 (44.81,48.95) | 47.75 (45.59,49.91) |  |
| 60–<150 minutes | 11.80 (10.77,12.84) | 13.75 (12.75,14.75) | 12.40 (11.32,13.48) |  |
| More than 150 minutes | 37.75 (35.91,39.59) | 39.37 (37.50,41.24) | 39.85 (37.97,41.73) |  |
| HEI |  |  |  | < 0.0001 |
| Quartile 1 (<40.396) | 27.00 (25.54,28.46) | 22.59 (21.23,23.94) | 24.17 (22.75,25.59) |  |
| Quartile 2 (40.396–49.668) | 25.21 (23.95,26.47) | 24.31 (23.04,25.57) | 24.60 (23.43,25.76) |  |
| Quartile 3 (49.669–59.543) | 24.15 (23.01,25.28) | 25.75 (24.53,26.97) | 24.53 (23.38,25.67) |  |
| Quartile 4 (>59.543) | 23.64 (22.21,25.07) | 27.35 (25.66,29.04) | 26.71 (24.90,28.51) |  |
| Work hours |  |  |  | 0.01 |
| 0 | 41.27 (39.69,42.85) | 40.91 (39.44,42.38) | 40.38 (38.66,42.10) |  |
| 1-34 | 13.56 (12.67,14.46) | 15.39 (14.26,16.52) | 15.69 (14.62,16.76) |  |
| 35-40 | 22.75 (21.53,23.98) | 20.80 (19.69,21.91) | 20.42 (19.06,21.78) |  |
| 41-55 | 15.04 (13.96,16.12) | 16.28 (15.23,17.33) | 16.21 (15.19,17.23) |  |
| More than 55 | 7.37 (6.65,8.10) | 6.62 (5.94,7.30) | 7.30 (6.50,8.10) |  |

| **Supplementary Table 10. Characteristics of participants with different proportion of breakfast energy intake** | | | | | | | | | | | | |  | | | |  |
| --- | --- | --- | --- | --- | --- | --- | --- | --- | --- | --- | --- | --- | --- | --- | --- | --- | --- |
| Characteristic | | | Proportion of Breakfast Energy Intake | | | | | | | | |  | |  |  |  |  |
|  |  |  | ≤13.3% | | | | >13.3%-23.9% | | >23.9% | | | *P* value | |  |  |  |  |
|  |  |  | (n = 11677) | | | | (n = 11658) | | (n = 11639) | | |  |  |  |  |  |  |
| Physical activity | | |  | | | |  | |  | | | < 0.0001 | |  |  |  |  |
| Inactive | | | 39.77 (37.65,41.88) | | | | 36.19 (34.39,38.00) | | 42.02 (40.08,43.96) | | |  | |  |  |  |  |
| Moderate | | | 23.08 (21.58,24.57) | | | | 25.77 (24.24,27.30) | | 23.27 (21.86,24.67) | | |  | |  |  |  |  |
| Vigorous | | | 7.74 (6.88,8.61) | | | | 6.90 (6.15,7.66) | | 7.20 (6.40,8.00) | | |  | |  |  |  |  |
| Both moderate and vigorous | | | 16.26 (14.91,17.61) | | | | 17.50 (16.04,18.96) | | 14.45 (13.04,15.86) | | |  | |  |  |  |  |
| Occupational status | | |  | | | |  | |  | | | < 0.0001 | |  |  |  |  |
| Working at a job or business | | | 63.01 (61.38,64.64) | | | | 58.75 (57.25,60.26) | | 54.60 (52.95,56.25) | | |  | |  |  |  |  |
| With a job or business but not at work | | | 2.48 (2.03,2.93) | | | | 2.45 (2.00,2.91) | | 2.56 (2.02,3.10) | | |  | |  |  |  |  |
| Looking for work | | | 3.70 (3.20,4.21) | | | | 2.66 (2.30,3.02) | | 3.23 (2.80,3.67) | | |  | |  |  |  |  |
| Not working | | | 30.74 (29.24,32.25) | | | | 36.08 (34.45,37.71) | | 39.55 (37.83,41.27) | | |  | |  |  |  |  |
| Family income | | |  | | | |  | |  | | | < 0.0001 | |  |  |  |  |
| Low income | | | 21.76 (20.17,23.35) | | | | 16.98 (15.73,18.23) | | 24.28 (22.71,25.86) | | |  | |  |  |  |  |
| Medium income | | | 31.82 (30.23,33.41) | | | | 32.84 (31.19,34.48) | | 34.04 (32.41,35.66) | | |  | |  |  |  |  |
| High income | | | 40.10 (38.00,42.20) | | | | 44.31 (41.95,46.66) | | 33.66 (31.64,35.67) | | |  | |  |  |  |  |
| BMI | | |  | | | |  | |  | | | < 0.0001 | |  |  |  |  |
| <18.5 kg/m2 | | | 1.91 (1.56,2.27) | | | | 1.60 (1.33,1.87) | | 1.33 (0.96,1.69) | | |  | |  |  |  |  |
| 18.5 to <25.0 kg/m^2^ | | | 30.56 (29.19,31.93) | | | | 28.71 (27.24,30.18) | | 26.88 (25.42,28.34) | | |  | |  |  |  |  |
| 25.0 to <30.0 kg/m^2^ | | | 30.62 (29.26,31.97) | | | | 33.67 (32.38,34.95) | | 33.64 (32.31,34.96) | | |  | |  |  |  |  |
| ≥30.0 kg/m^2^ | | | 36.91 (35.30,38.51) | | | | 36.03 (34.53,37.52) | | 38.16 (36.76,39.55) | | |  | |  |  |  |  |
| Total energy intakes | | |  | | | |  | |  | | | < 0.0001 | |  |  |  |  |
| Quartile 1 (<1458 kcal) | | | 21.28 (20.03,22.53) | | | | 19.04 (17.91,20.17) | | 27.16 (25.87,28.45) | | |  | |  |  |  |  |
| Quartile 2 (1458–1916.5 kcal) | | | 22.75 (21.60,23.90) | | | | 26.11 (25.01,27.21) | | 27.02 (25.83,28.22) | | |  | |  |  |  |  |
| Quartile 3 (1917–2491 kcal) | | | 25.78 (24.64,26.93) | | | | 27.28 (26.08,28.48) | | 24.94 (23.70,26.19) | | |  | |  |  |  |  |
| Quartile 4 (>2491 kcal) | | | 30.19 (28.81,31.56) | | | | 27.57 (26.43,28.70) | | 20.87 (19.72,22.02) | | |  | |  |  |  |  |
| Duration of recreational physical activity | | |  | | | |  | |  | | | < 0.0001 | |  |  |  |  |
| Less than 60 minutes | | | 49.04 (46.80,51.29) | | | | 45.04 (43.01,47.07) | | 51.54 (49.46,53.61) | | |  | |  |  |  |  |
| 60–<150 minutes | | | 12.59 (11.56,13.62) | | | | 13.45 (12.40,14.49) | | 11.78 (10.87,12.69) | | |  | |  |  |  |  |
| More than 150 minutes | | | 38.37 (36.43,40.31) | | | | 41.51 (39.64,43.38) | | 36.69 (34.82,38.55) | | |  | |  |  |  |  |
| HEI | | |  | | | |  | |  | | | < 0.0001 | |  |  |  |  |
| Quartile 1 (<40.396) | | | 29.89 (28.53,31.24) | | | | 20.89 (19.53,22.24) | | 22.27 (20.96,23.58) | | |  | |  |  |  |  |
| Quartile 2 (40.396–49.668) | | | 27.76 (26.54,28.97) | | | | 23.66 (22.55,24.77) | | 22.05 (20.82,23.28) | | |  | |  |  |  |  |
| Quartile 3 (49.669–59.543) | | | 23.67 (22.50,24.84) | | | | 25.72 (24.68,26.77) | | 25.14 (23.92,26.37) | | |  | |  |  |  |  |
| Quartile 4 (>59.543) | | | 18.68 (17.27,20.10) | | | | 29.73 (28.17,31.30) | | 30.54 (28.90,32.17) | | |  | |  |  |  |  |
| Work hours | | |  | | | |  | |  | | | < 0.0001 | |  |  |  |  |
| 0 | | | 36.96 (35.33,38.59) | | | | 41.23 (39.71,42.74) | | 45.39 (43.74,47.03) | | |  | |  |  |  |  |
| 1-34 | | | 15.44 (14.39,16.49) | | | | 15.38 (14.29,16.47) | | 13.65 (12.70,14.60) | | |  | |  |  |  |  |
| 35-40 | | | 22.33 (21.16,23.51) | | | | 20.72 (19.47,21.97) | | 20.64 (19.50,21.77) | | |  | |  |  |  |  |
| 41-55 | | | 17.55 (16.49,18.61) | | | | 15.83 (14.85,16.81) | | 13.72 (12.67,14.77) | | |  | |  |  |  |  |
| More than 55 | | | 7.72 (6.97,8.47) | | | | 6.84 (6.04,7.64) | | 6.61 (5.92,7.30) | | |  | |  |  |  |  |
| **Supplementary Table 11. Characteristics of participants with different breakfast time** | | | | | | | | | | | |  | |  |  |  |  |
| Characteristic | | Breakfast time | | | | | | | | | |  | | | |  |  |
|  |  | Before 7:00 AM | | | Between 7:00 and before 9:00 AM | | | | | 9:00 AM or later | | *P* value | | | |  |  |
|  |  | (n = 4554) | | | (n = 16244) | | | | | (n = 11350) | |  |  |  |  |  |  |
| Physical activity | |  | | |  | | | | |  | | 0.002 | | | |  |  |
| Inactive | | 38.68 (35.76,41.61) | | | 36.66 (34.99,38.34) | | | | | 41.38 (39.48,43.28) | |  | | | |  |  |
| Moderate | | 24.37 (21.94,26.79) | | | 26.36 (25.12,27.60) | | | | | 21.41 (20.31,22.50) | |  | | | |  |  |
| Vigorous | | 7.06 (5.96,8.15) | | | 6.85 (6.22,7.49) | | | | | 7.75 (6.89,8.62) | |  | | | |  |  |
| Both moderate and vigorous | | 15.30 (13.49,17.12) | | | 16.59 (15.24,17.93) | | | | | 16.88 (15.48,18.29) | |  | | | |  |  |
| Occupational status | |  | | |  | | | | |  | | < 0.0001 | | | |  |  |
| Working at a job or business | | 70.86 (68.83,72.89) | | | 58.19 (56.75,59.62) | | | | | 54.38 (52.84,55.92) | |  | | | |  |  |
| With a job or business but not at work | | 1.72 (1.24,2.21) | | | 2.55 (2.10,2.99) | | | | | 2.82 (2.28,3.36) | |  | | | |  |  |
| Looking for work | | 1.46 (1.01,1.91) | | | 2.30 (2.00,2.59) | | | | | 4.81 (4.24,5.38) | |  | | | |  |  |
| Not working | | 25.95 (23.99,27.90) | | | 36.88 (35.41,38.35) | | | | | 37.97 (36.40,39.53) | |  | | | |  |  |
| Family income | |  | | |  | | | | |  | | < 0.0001 | | | |  |  |
| Low income | | 14.47 (13.02,15.92) | | | 16.97 (15.84,18.09) | | | | | 26.86 (25.19,28.53) | |  | | | |  |  |
| Medium income | | 30.93 (28.46,33.41) | | | 32.79 (31.20,34.38) | | | | | 33.69 (31.68,35.69) | |  | | | |  |  |
| High income | | 48.49 (45.41,51.57) | | | 43.88 (41.86,45.90) | | | | | 32.20 (29.98,34.42) | |  | | | |  |  |
| BMI | |  | | |  | | | | |  | | < 0.0001 | | | |  |  |
| <18.5 kg/m2 | | 1.53 (0.97,2.08) | | | 1.20 (0.97,1.43) | | | | | 2.25 (1.88,2.63) | |  | | | |  |  |
| 18.5 to <25.0 kg/m^2^ | | 24.93 (22.81,27.05) | | | 28.09 (26.86,29.31) | | | | | 31.46 (30.04,32.89) | |  | | | |  |  |
| 25.0 to <30.0 kg/m^2^ | | 35.84 (33.83,37.84) | | | 34.32 (33.15,35.48) | | | | | 29.75 (28.54,30.95) | |  | | | |  |  |
| ≥30.0 kg/m^2^ | | 37.71 (35.59,39.83) | | | 36.40 (35.09,37.71) | | | | | 36.54 (34.91,38.17) | |  | | | |  |  |
| Total energy intakes | |  | | |  | | | | |  | | < 0.0001 | | | |  |  |
| Quartile 1 (<1458 kcal) | | 19.03 (17.36,20.71) | | | 21.08 (19.94,22.23) | | | | | 23.00 (21.92,24.08) | |  | | | |  |  |
| Quartile 2 (1458–1916.5 kcal) | | 23.85 (22.05,25.64) | | | 25.81 (24.88,26.74) | | | | | 25.43 (24.22,26.64) | |  | | | |  |  |
| Quartile 3 (1917–2491 kcal) | | 26.48 (24.70,28.26) | | | 27.20 (26.21,28.19) | | | | | 24.87 (23.68,26.06) | |  | | | |  |  |
| Quartile 4 (>2491 kcal) | | 30.64 (28.62,32.66) | | | 25.90 (24.79,27.02) | | | | | 26.70 (25.74,27.67) | |  | | | |  |  |
| Duration of recreational physical activity | |  | | |  | | | | |  | | < 0.001 | | | |  |  |
| Less than 60 minutes | | 48.13 (45.48,50.78) | | | 45.96 (44.15,47.77) | | | | | 50.28 (48.15,52.41) | |  | | | |  |  |
| 60–<150 minutes | | 12.19 (10.39,13.99) | | | 13.63 (12.81,14.46) | | | | | 11.82 (10.76,12.88) | |  | | | |  |  |
| More than 150 minutes | | 39.69 (36.90,42.47) | | | 40.41 (38.69,42.13) | | | | | 37.90 (36.01,39.79) | |  | | | |  |  |
| HEI | |  | | |  | | | | |  | | < 0.0001 | | | |  |  |
| Quartile 1 (<40.396) | | 23.15 (21.06,25.25) | | | 20.82 (19.60,22.05) | | | | | 27.13 (25.76,28.51) | |  | | | |  |  |
| Quartile 2 (40.396–49.668) | | 24.68 (22.80,26.55) | | | 23.52 (22.40,24.65) | | | | | 25.44 (24.31,26.57) | |  | | | |  |  |
| Quartile 3 (49.669–59.543) | | 24.67 (22.89,26.46) | | | 25.51 (24.45,26.56) | | | | | 24.77 (23.53,26.01) | |  | | | |  |  |
| Quartile 4 (>59.543) | | 27.49 (24.95,30.04) | | | 30.15 (28.55,31.74) | | | | | 22.66 (21.21,24.11) | |  | | | |  |  |
| Work hours | |  | | |  | | | | |  | | < 0.0001 | | | |  |  |
| 0 | | 29.14 (27.12,31.17) | | | 41.77 (40.34,43.20) | | | | | 45.62 (44.08,47.16) | |  | | | |  |  |
| 1-34 | | 12.24 (10.78,13.70) | | | 14.74 (13.76,15.71) | | | | | 16.17 (15.10,17.25) | |  | | | |  |  |
| 35-40 | | 24.56 (22.80,26.32) | | | 20.79 (19.84,21.75) | | | | | 20.64 (19.44,21.83) | |  | | | |  |  |
| 41-55 | | 22.64 (20.63,24.66) | | | 16.07 (15.20,16.94) | | | | | 12.23 (11.35,13.12) | |  | | | |  |  |
| More than 55 | | 11.41 (9.88,12.95) | | | 6.63 (6.10, 7.16) | | | | | 5.34 (4.70, 5.97) | |  | | | |  |  |
| **Supplementary Table 12. Characteristics of participants with different proportion of lunch energy intake** | | | | | | | | | | | | | | | |  |  |
| Characteristic | | | Proportion of Lunch Energy Intake | | | | | | |  | | | |  |  |  |  |
|  |  |  | ≤17.1% | | >17.1%-30.5% | | >30.5% | | | *P* value | | | |  |  |  |  |
|  |  |  | (n = 11659) | | (n = 11675) | | (n = 11640) | | |  |  |  |  |  |  |  |  |
| Physical activity | | |  | |  | |  | | | < 0.0001 | | | |  |  |  |  |
| Inactive | | | 43.32 (41.37,45.26) | | 35.30 (33.25,37.34) | | 39.34 (37.65,41.04) | | |  | | | |  |  |  |  |
| Moderate | | | 23.23 (21.73,24.72) | | 25.59 (24.23,26.94) | | 23.30 (21.72,24.88) | | |  | | | |  |  |  |  |
| Vigorous | | | 6.37 (5.70,7.05) | | 7.10 (6.36,7.84) | | 8.35 (7.47,9.24) | | |  | | | |  |  |  |  |
| Both moderate and vigorous | | | 13.94 (12.80,15.08) | | 18.72 (17.14,20.29) | | 15.57 (14.24,16.90) | | |  | | | |  |  |  |  |
| Occupational status | | |  | |  | |  | | | < 0.0001 | | | |  |  |  |  |
| Working at a job or business | | | 55.07 (53.67,56.47) | | 60.10 (58.60,61.59) | | 61.88 (60.34,63.42) | | |  | | | |  |  |  |  |
| With a job or business but not at work | | | 2.19 (1.79,2.58) | | 2.66 (2.06,3.26) | | 2.59 (2.14,3.04) | | |  | | | |  |  |  |  |
| Looking for work | | | 3.65 (3.17,4.12) | | 2.87 (2.47,3.28) | | 3.13 (2.68,3.58) | | |  | | | |  |  |  |  |
| Not working | | | 39.00 (37.54,40.45) | | 34.33 (32.86,35.81) | | 32.35 (30.88,33.83) | | |  | | | |  |  |  |  |
| Family income | | |  | |  | |  | | | < 0.0001 | | | |  |  |  |  |
| Low income | | | 24.65 (22.96,26.34) | | 17.63 (16.32,18.94) | | 20.48 (19.07,21.90) | | |  | | | |  |  |  |  |
| Medium income | | | 34.45 (32.86,36.03) | | 31.51 (29.77,33.25) | | 32.69 (31.14,34.24) | | |  | | | |  |  |  |  |
| High income | | | 33.59 (31.43,35.75) | | 44.86 (42.53,47.19) | | 40.13 (38.05,42.22) | | |  | | | |  |  |  |  |
| BMI | | |  | |  | |  | | | 0.01 | | | |  |  |  |  |
| <18.5 kg/m2 | | | 1.39 (1.08,1.71) | | 1.88 (1.52,2.24) | | 1.60 (1.26,1.93) | | |  | | | |  |  |  |  |
| 18.5 to <25.0 kg/m^2^ | | | 28.68 (27.22,30.14) | | 30.05 (28.45,31.65) | | 27.76 (26.27,29.26) | | |  | | | |  |  |  |  |
| 25.0 to <30.0 kg/m^2^ | | | 33.24 (31.69,34.78) | | 32.58 (31.30,33.87) | | 31.90 (30.57,33.22) | | |  | | | |  |  |  |  |
| ≥30.0 kg/m^2^ | | | 36.69 (35.15,38.24) | | 35.49 (34.00,36.98) | | 38.74 (37.04,40.44) | | |  | | | |  |  |  |  |
| Total energy intakes | | |  | |  | |  | | | < 0.0001 | | | |  |  |  |  |
| Quartile 1 (<1458 kcal) | | | 26.06 (24.78,27.35) | | 18.49 (17.52,19.45) | | 22.37 (21.19,23.56) | | |  | | | |  |  |  |  |
| Quartile 2 (1458–1916.5 kcal) | | | 24.12 (22.95,25.30) | | 25.43 (24.34,26.53) | | 25.80 (24.75,26.84) | | |  | | | |  |  |  |  |
| Quartile 3 (1917–2491 kcal) | | | 23.77 (22.70,24.83) | | 27.78 (26.46,29.10) | | 26.41 (25.25,27.56) | | |  | | | |  |  |  |  |
| Quartile 4 (>2491 kcal) | | | 26.05 (24.85,27.24) | | 28.30 (27.19,29.41) | | 25.42 (24.23,26.61) | | |  | | | |  |  |  |  |
| Duration of recreational physical activity | | |  | |  | |  | | | < 0.0001 | | | |  |  |  |  |
| Less than 60 minutes | | | 53.06 (51.06,55.05) | | 43.69 (41.46,45.92) | | 48.91 (46.90,50.91) | | |  | | | |  |  |  |  |
| 60–<150 minutes | | | 11.36 (10.30,12.42) | | 13.34 (12.36,14.31) | | 13.17 (12.26,14.07) | | |  | | | |  |  |  |  |
| More than 150 minutes | | | 35.59 (33.78,37.39) | | 42.97 (40.94,45.00) | | 37.93 (36.05,39.81) | | |  | | | |  |  |  |  |
| HEI | | |  | |  | |  | | | < 0.0001 | | | |  |  |  |  |
| Quartile 1 (<40.396) | | | 26.31 (24.92,27.70) | | 20.99 (19.67,22.30) | | 26.73 (25.31,28.15) | | |  | | | |  |  |  |  |
| Quartile 2 (40.396–49.668) | | | 25.27 (24.08,26.45) | | 23.44 (22.14,24.74) | | 25.53 (24.17,26.89) | | |  | | | |  |  |  |  |
| Quartile 3 (49.669–59.543) | | | 24.70 (23.62,25.79) | | 25.87 (24.53,27.21) | | 23.78 (22.63,24.94) | | |  | | | |  |  |  |  |
| Quartile 4 (>59.543) | | | 23.72 (22.14,25.31) | | 29.70 (28.00,31.40) | | 23.96 (22.48,25.43) | | |  | | | |  |  |  |  |
| Work hours | | |  | |  | |  | | | < 0.0001 | | | |  |  |  |  |
| 0 | | | 44.89 (43.48,46.30) | | 39.89 (38.39,41.38) | | 38.11 (36.57,39.65) | | |  | | | |  |  |  |  |
| 1-34 | | | 14.69 (13.69,15.69) | | 15.20 (14.14,16.27) | | 14.83 (13.66,16.01) | | |  | | | |  |  |  |  |
| 35-40 | | | 19.51 (18.39,20.63) | | 21.89 (20.66,23.12) | | 22.26 (21.22,23.31) | | |  | | | |  |  |  |  |
| 41-55 | | | 13.64 (12.78,14.51) | | 16.54 (15.54,17.53) | | 17.20 (16.08,18.32) | | |  | | | |  |  |  |  |
| More than 55 | | | 7.27 (6.53,8.02) | | 6.48 (5.74,7.23) | | 7.59 (6.88,8.30) | | |  | | | |  |  |  |  |

**Supplementary Table 13. Association between energy intake and depressive symptoms at different time periods**

| Exposure^a^ | Model^b^ | Cut points | No.^c^ | OR (95% CI) | *P* value | *P* for nonlinearity |
| --- | --- | --- | --- | --- | --- | --- |
| Proportion of early morning (5-9 AM) energy intake | Crude model | <21% | 28498 | **0.70 (0.63, 0.77)** | **<0.001** | **<0.001** |
|  |  | >21% | 6476 | **1.40 (1.18, 1.67)** | **<0.001** |  |
|  | Adjusted model | <23% | 29774 | **0.84 (0.75, 0.94)** | **0.002** | **<0.001** |
|  |  | >23% | 5200 | 1.17 (0.98, 1.41) | 0.082 |  |
| Proportion of late morning (9-12 PM) energy intake | Crude model | <9% | 15158 | 0.84 (0.65, 1.09) | 0.194 | **0.037** |
|  |  | >9% | 19816 | **1.10 (1.02, 1.18)** | **0.01** |  |
|  | Adjusted model | per 10% increase | 34974 | 1.03 (0.98, 1.08) | 0.207 | 0.527 |
| Proportion of midday (12 PM-1 PM) energy intake | Crude model | <29% | 31340 | **0.83 (0.78, 0.88)** | **<0.001** | **0.002** |
|  |  | >29% | 3634 | 1.03 (0.85, 1.23) | 0.78 |  |
|  | Adjusted model | per 10% increase | 34974 | **0.85 (0.75, 0.96)** | **0.01** | 0.632 |
| Proportion of afternoon (1-6 PM) energy intake | Crude model | <36% | 23566 | 1.00 (0.94, 1.06) | 0.898 | **0.003** |
|  |  | >36% | 11408 | **1.13 (1.05, 1.22)** | **0.001** |  |
|  | Adjusted model | <42% | 26930 | 0.97 (0.92, 1.03) | 0.361 | 0.072 |
|  |  | >42% | 8044 | 1.01 (0.92, 1.09) | 0.902 |  |
| Proportion of evening (6-8 PM) energy intake | Crude model | <36% | 28431 | **0.88 (0.84, 0.92)** | **<0.001** | **<0.001** |
|  |  | >36% | 6543 | 1.10 (0.97, 1.24) | 0.123 |  |
|  | Adjusted model | <39% | 29753 | 0.98 (0.93, 1.03) | 0.064 | 0.621 |
|  |  | >39% | 5221 | 1.13 (0.99, 1.28) | 0.39 |  |
|  |  | per 10% increase | 34974 | 0.97 (0.94, 1.01) | 0.167 |  |
| Proportion of evening (8-11 PM) energy intake | Crude model | per 10% increase | 34974 | **1.06 (1.03, 1.10)** | **<0.001** | 0.883 |
|  | Adjusted model | per 10% increase | 34974 | 1.04 (1.00, 1.08) | 0.055 | 0.434 |
| Proportion of overnight (11 PM-5 AM) energy intake | Crude model | per 10% increase | 34974 | **1.22 (1.16, 1.29)** | **<0.001** |  |
|  |  | Dichotomy |  |  |  |  |
|  |  | No energy intake | 27885 | Reference (1) |  | **<0.001** |
|  |  | Taking energy at night | 7089 | **1.65 (1.45, 1.86)** | **<0.001** |  |
|  | Adjusted model | per 10% increase | 34974 | **1.17 (1.10, 1.24)** | **<0.001** |  |
|  |  | Dichotomy |  |  |  |  |
|  |  | No energy intake | 27885 | Reference (1) |  | **<0.001** |
|  |  | Taking energy at night | 7089 | **1.42 (1.23, 1.63)** | **<0.001** |  |

Abbreviations: OR, odds ratio; CI, confidence interval.

^a^Detailed definitions of the exposures are provided in eTable 5 in Supplement.

^b^Crude Model were unadjusted. Adjusted model were adjusted for age, sex, race and ethnicity, education level, marital status, family income, body mass index, smoking status, drinking status, physical activity, comorbid condition, sleep duration, total energy intakes, Healthy Eating Index and dietary recall day of the week.

**Supplementary Table 14. Association between meals and depressive symptoms**

| Exposure^a^ | No. | Crude model^b^ | | Adjusted model^c^ | |
| --- | --- | --- | --- | --- | --- |
|  |  | OR (95% CI) | *P* value | OR (95% CI) | *P* value |
| Eatting breakfast |  |  |  |  |  |
| Skipping breakfast at both dietary recalls | 1989 | Reference (1) | - | Reference (1) | - |
| Participanted in one dietary recall and reported skipping breakfast | 837 | 0.99 (0.67, 1.48) | 0.979 | 1.18 (0.55, 2.57) | 0.664 |
| Participanted in both dietary recall and reported eatting breakfast only once | 5169 | **0.72 (0.57, 0.91)** | **0.006** | 0.91 (0.71, 1.16) | 0.434 |
| Participanted in one dietary recall and reported eatting breakfast | 3323 | **0.46 (0.37, 0.59)** | **<0.001** | 0.69 (0.36, 1.35) | 0.274 |
| Both dietary recalls reported eating breakfast | 23656 | **0.47 (0.39, 0.57)** | **<0.001** | **0.79 (0.63, 0.99)** | **0.041** |
| Eatting lunch |  |  |  |  |  |
| Skipping lunch at both dietary recalls | 3107 | Reference (1) | - | Reference (1) | - |
| Participanted in one dietary recall and reported skipping lunch | 1112 | **0.60 (0.44, 0.82)** | **0.001** | 0.63 (0.31, 1.29) | 0.204 |
| Participanted in both dietary recall and reported eatting lunch only once | 8191 | **0.68 (0.56, 0.81)** | **<0.001** | **0.78 (0.65, 0.94)** | **0.011** |
| Participanted in one dietary recall and reported eatting lunch | 3048 | **0.52 (0.41, 0.65)** | **<0.001** | 0.70 (0.35, 1.41) | 0.316 |
| Both dietary recalls reported eating lunch | 19516 | **0.40 (0.34, 0.48)** | **<0.001** | **0.64 (0.53, 0.77)** | **<0.001** |
| Eatting dinner |  |  |  |  |  |
| Skipping dinner at both dietary recalls | 807 | Reference (1) | - | Reference (1) | - |
| Participanted in one dietary recall and reported skipping dinner | 543 | 0.84 (0.49, 1.43) | 0.516 | 0.93 (0.36, 2.40) | 0.883 |
| Participanted in both dietary recall and reported eatting dinner only once | 4103 | 1.04 (0.73, 1.48) | 0.813 | 1.26 (0.85, 1.87) | 0.242 |
| Participanted in one dietary recall and reported eatting dinner | 3617 | **0.70 (0.50, 0.98)** | **0.036** | 0.97 (0.46, 2.03) | 0.925 |
| Both dietary recalls reported eating dinner | 25904 | **0.63 (0.46, 0.87)** | **0.005** | 0.98 (0.67, 1.42) | 0.91 |

Abbreviations: OR, odds ratio; CI, confidence interval.

^a^Detailed definitions of the exposures are provided in eTable 5 in Supplement.

^b^Crude Model were unadjusted.

^c^Adjusted model were adjusted for age, sex, race and ethnicity, education level, marital status, family income, body mass index, smoking status, drinking status, physical activity, comorbid condition, sleep duration, total energy intakes, Healthy Eating Index and dietary recall day of the week.

**eReferences**

1. Agricultural Research Service. WWEIA Data Tables : USDA ARS. 2022. Accessed February 28, 2023. https://www.ars.usda.gov/northeast-area/beltsville-md-bhnrc/beltsville-human-nutrition-research-center/food-surveys-research-group/docs/wweia-data-tables/

2. Krebs-Smith SM, Pannucci TE, Subar AF, et al. Update of the Healthy Eating Index: HEI-2015. *J Acad Nutr Diet*. 2018;118(9):1591-1602. doi:10.1016/j.jand.2018.05.021

3. Paans NPG, Gibson-Smith D, Bot M, et al. Depression and eating styles are independently associated with dietary intake. *Appetite*. 2019;134:103-110. doi:10.1016/j.appet.2018.12.030

4. Sangouni AA, Beigrezaei S, Akbarian S, et al. Association between dietary behaviors and depression in adolescent girls. *BMC Public Health*. 2022;22(1):1169. doi:10.1186/s12889-022-13584-0

5. Sousa KT de, Marques ES, Levy RB, Azeredo CM. Food consumption and depression among Brazilian adults: results from the Brazilian National Health Survey, 2013. *Cad Saude Publica*. 2019;36(1):e00245818. doi:10.1590/0102-311X00245818

6. Ansu Baidoo VY, Zee PC, Knutson KL. Racial and Ethnic Differences in Eating Duration and Meal Timing: Findings from NHANES 2011–2018. *Nutrients*. 2022;14(12):2428. doi:10.3390/nu14122428

7. Wilson JE, Blizzard L, Gall SL, et al. An eating pattern characterised by skipped or delayed breakfast is associated with mood disorders among an Australian adult cohort. *Psychol Med*. 2020;50(16):2711-2721. doi:10.1017/S0033291719002800

8. Barrera CM, Moore LV, Perrine CG, Hamner HC. Number of Eating Occasions and Source of Foods and Drinks Among Young Children in the United States: NHANES, 2009^−^2014. *Nutrients*. 2019;11(4):897. doi:10.3390/nu11040897

9. Taillie LS, Afeiche MC, Eldridge AL, Popkin BM. Increased Snacking and Eating Occasions Are Associated with Higher Energy Intake among Mexican Children Aged 2–13 Years. *J Nutr*. 2015;145(11):2570-2577. doi:10.3945/jn.115.213165

10. Zuercher JL, Wagstaff DA, Kranz S. Associations of food group and nutrient intake, diet quality, and meal sizes between adults and children in the same household: a cross-sectional analysis of U.S. households. *Nutr J*. 2011;10:131. doi:10.1186/1475-2891-10-131

11. Suzuki A, Sakurazawa H, Fujita T, Akamatsu R. Overeating at dinner time among Japanese workers: Is overeating related to stress response and late dinner times? *Appetite*. 2016;101:8-14. doi:10.1016/j.appet.2016.02.145

12. Zahedi H, Djalalinia S, Sadeghi O, et al. Breakfast consumption and mental health: a systematic review and meta-analysis of observational studies. *Nutr Neurosci*. 2022;25(6):1250-1264. doi:10.1080/1028415X.2020.1853411

13. Fulkerson JA, Sherwood NE, Perry CL, Neumark-Sztainer D, Story M. Depressive symptoms and adolescent eating and health behaviors: a multifaceted view in a population-based sample. *Prev Med*. 2004;38(6):865-875. doi:10.1016/j.ypmed.2003.12.028

14. Yun H, Kim DW, Lee EJ, Jung J, Yoo S. Analysis of the Effects of Nutrient Intake and Dietary Habits on Depression in Korean Adults. *Nutrients*. 2021;13(4):1360. doi:10.3390/nu13041360

15. Kwak Y, Kim Y. Association between mental health and meal patterns among elderly Koreans. *Geriatr Gerontol Int*. 2018;18(1):161-168. doi:10.1111/ggi.13106

16. Tajik E, Latiffah AL, Awang H, et al. Unhealthy diet practice and symptoms of stress and depression among adolescents in Pasir Gudang, Malaysia. *Obes Res Clin Pract*. 2016;10(2):114-123. doi:10.1016/j.orcp.2015.06.001
